# Marine actinobacterium *Streptomyces vinaceusdrappus *mediated nano-selenium: biosynthesis and biomedical activities

**DOI:** 10.1186/s12906-025-05073-9

**Published:** 2025-09-17

**Authors:** Ahmed Ghareeb, Amr Fouda, Rania M. Kishk, Waleed M. El Kazzaz

**Affiliations:** 1 Botany and Microbiology Department, Faculty of Science, Suez Canal University, Ismailia, 41522 Egypt; 2 Botany and Microbiology Department, Faculty of Science, Al-Azhar University, Nasr City, 11884 Cairo Egypt; 3 Microbiology and Immunology Department, Faculty of Medicine, Suez Canal University, Ismailia, 41522 Egypt

**Keywords:** Biogenic synthesis, Marine actinobacteria, Selenium nanoparticles, Characterizations, Biomedical nanomaterials

## Abstract

**Background:**

Biogenic Selenium nanoparticles (Se-NPs) have been receiving more attention, primarily due to their remarkable biomedical potential. This study was designed to biosynthesize Se-NPs using marine actinobacterium *Streptomyces vinaceusdrappus* AMG31 metabolites and comprehensively evaluate their multifunctional biomedical applications.

**Methods:**

The actinobacterial biofabricated Se-NPs were characterized by UV-vis spectroscopy, FT-IR, XRD, TEM, EDX, DLS, and zeta potential. Se-NPs were then evaluated for their biomedical applications, including antioxidant (DPPH, ABTS, TAC, and FRAP assays), wound healing (scratch assay on HFB4 cell line), hemocompatibility, anticancer (crystal violet viability assay), antidiabetic (α-amylase and α-glucosidase inhibition), antimicrobial (agar well diffusion and MIC/MBC/MFC determination), antibiofilm, and anti-inflammatory properties (COX-enzymes in vitro inhibition).

**Results:**

Formed Se-NPs exhibited crystalline, stable, and spherical structures with sizes ranging from 20 to 80 nm and a negative zeta potential of -37.9 mV. Se-NPs exhibited potent antioxidant activity, achieving 86.7% DPPH scavenging, 84.6% ABTS scavenging, a TAC value of 965.967 µg/mg ascorbic acid equivalent, and a FRAP value of 727.667 µg/mg ascorbic acid equivalent. Se-NPs promoted enhanced wound healing, with a wound closure percentage of 73.6% at 209.87 µg mL^–1^. Se-NPs displayed selective cytotoxicity towards Caco-2 (IC_50_ = 102.5 µg mL^–1^) and PANC-1 (IC_50_ = 100.4 µg mL^–1^) cancer cells while exhibiting minimal toxicity against normal WI-38 fibroblasts (IC_50_ = 419.7 µg mL^–1^). Furthermore, Se-NPs demonstrated concentration-dependent inhibition of α-amylase (IC_50_ = 59.8 µg mL^–1^) and α-glucosidase (IC_50_ = 19.3 µg mL^–1^), suggesting antidiabetic potential. Nano-selenium exhibited promising antifungal activity against *Aspergillus niger* (MIC = 25 µg mL^–1^, MFC = 200 µg mL^–1^), *Penicillium glabrum* (MIC = 50 µg mL^–1^, MFC = 200 µg mL^–1^), *Mucor circinelloides* (MIC = 100 µg mL^–1^, MFC = 300 µg mL^–1^), *Trichoderma harzianum* (MIC = 100 µg mL^–1^, MFC = 200 µg mL^–1^), and *Candida albicans* (MIC = 12.5 µg mL^–1^, MFC = 50 µg mL^–1^ and 95.4% biofilm inhibition). Moreover, nanostructure showed antibacterial activity most effectively against *Enterococcus faecalis* (MIC = 25 µg mL^–1^, MBC = 50 µg mL^–1^) and *Pseudomonas aeruginosa* (MIC = 50 µg mL^–1^, MBC = 50 µg mL^–1^) with over 90% inhibition of biofilm formation. Finally, the nano-selenium structure showed potent inhibitory action against COX-1 (IC_50_ = 30.9 µg mL^–1^) and COX-2 (IC_50_ = 46.6 µg mL^–1^).

**Conclusion:**

The obtained results confirmed the efficacy of marine actinobacterium *S. vinaceusdrappus* metabolites to fabricate Se-NPs which exhibits broad spectrum biomedical activities including antioxidant, anticancer, antidiabetics, antimicrobial, antibiofilm, anti-inflammatory, and wound healing activities.

**Supplementary Information:**

The online version contains supplementary material available at 10.1186/s12906-025-05073-9.

## Introduction

The emergence of various medical challenges, such as multidrug resistant microbes, wound healing, cancer propagation, inflammatory and diabetic spread, etc., has led to traditional medication resistance. This development has recently caused significant public health threats. Such an upward trend of resistance rates has raised alarm bells in healthcare facilities to develop new active compounds to combat these challenges [1, 2]. Nanoparticles such as metal and metal oxide (such as Ag, Ti, Cu, Zn, or their oxide forms) display antimicrobial properties and have been extensively researched [3]. Nevertheless, certain research has indicated that certain metal nanoparticles yield toxic effects [4]. In this regard, nanoscale selenium and tellurium metalloids have been of increasing interest for their unique properties owing to their partially metal and non-metal character, which could provide room for numerous applications [5].

Selenium (Se) is a semiconductor with multiple industrial applications, which include photovoltaic cells and xenography [6]. Se is one of the trace elements that is deemed crucial in maintaining human health. It assists in cellular defense mechanisms, improves thyroid functions, and modulates immunity while also having potent anticancer and antimicrobial activities [7]. Nevertheless, excessive doses of it can cause adverse effects [8]. Se-NPs are gradually garnering worldwide attention, as they show enhanced efficacy in inhibiting oxidative damage in comparison with other cases of Se-based compounds owing to their considerable biological efficiency, high bioavailability, and minimal toxicity [9].

Selenium nanoparticles (Se-NPs) synthesis encompasses three primary approaches: chemical methods (chemical reduction using ascorbic acid, hydrazine, or sodium borohydride; sol-gel techniques; and hydrothermal processes), physical methods (laser ablation, vapor deposition, and microwave-assisted synthesis), and biological employs bacteria, fungi, plant extracts, algae, and yeast to create highly biocompatible particles with natural stabilizers. Chemical and physical methods, while offering controlled synthesis, have significant drawbacks. Chemical approaches use toxic precursors requiring extensive purification and often produce particles with harmful stabilizers. Physical techniques demand specialized, energy-intensive equipment with high costs and frequently yield broad size distributions with limited output. Both generate hazardous waste, use environmentally damaging chemicals, and produce nanoparticles with poorer biocompatibility than biogenic alternatives, restricting their therapeutic potential despite more consistent production [3].

Biogenic Se-NPs exhibit superior long-term stability due to their colloidal nature and polydispersity [10]. They are maintained by organic molecules and natural capping agents derived from biological sources that create biocompatible structures without requiring synthetic chemicals [11]. The eco-friendly approach encounters reproducibility challenges stemming from batch-to-batch metabolite variations that affect particle properties, a double-edged characteristic providing application diversity while complicating standardization [12]. Scaling up demands solutions for vessel-dependent reaction kinetics, biomolecule-selenium ratio consistency in larger volumes, and advanced monitoring technologies. Emerging continuous-flow bioreactor systems with precise nutrient control represent significant advancement potential for industrial implementation [13].

Se-NPs suppress cancer cells without harming normal tissue through reactive oxygen species (ROS) disruption and mitochondrial damage, while starving tumors by blocking blood vessel formation [14]. These particles rupture microbial membranes and shut down essential metabolic pathways involving phosphoglucose isomerase and pyruvate dehydrogenase. Their dual role as ROS neutralizers and enzyme boosters, particularly glutathione peroxidase and superoxide dismutase, makes them effective against oxidative damage [15]. Beyond these roles, Se-NPs reduce inflammation by controlling cytokine production, accelerate wound repair, and boost insulin effectiveness in diabetics [16] and shield neurons from deterioration in Alzheimer’s and Parkinson’s disease by tackling brain inflammation and oxidative assault [17].

Actinomycetes, a diverse group of filamentous bacteria, play a pivotal role in antibiotic discovery, with *Streptomyces* constituting the largest genus within actinobacteria, encompassing 826 species and representing approximately 75% of the total 7600 metabolites secreted by actinobacteria. These microorganisms, particularly those from the genus *Streptomyces*, are responsible for producing about 70% of all known antibiotics, including macrolides, aminoglycosides, and rifamycins, while approximately 45% of bioactive compounds derived from microbes originate from actinomycetes [1]. Other significant genera like *Micromonospora* contribute additional antibiotics such as gentamicin and fortimicins, highlighting the taxonomic diversity within this group [18].

*Streptomyces vinaceusdrappus*, belonging to the Actinobacteria phylum and Streptomycetaceae family, produces the antibiotic streptonigrin and exhibits remarkable therapeutic potential through its secondary metabolites that demonstrate strong antimicrobial activity against multidrug-resistant pathogens and cytotoxic effects on cancer cells through mechanisms involving DNA intercalation and topoisomerase inhibition [19]. This species plays vital roles in nutrient cycling through the decomposition of organic matter and the production of geosmin, the compound responsible for the characteristic earthy odor after rain [20]. The metabolites produced by actinomycetes, including tetracyclines and macrolides, salinosporamide A, and calicheamicin, have demonstrated anticancer effects against various cell lines, such as breast (MCF-7) and liver (HepG2) cancers [21], while yielding important antiviral agents [22, 23]. Recent advances in genome mining and genetic manipulation have enhanced the discovery and activation of cryptic secondary metabolite gene clusters in Streptomyces, facilitating the development of new compounds to combat antibiotic resistance and develop novel therapeutic strategies [24].

The foundation for exploring selenium’s wide-ranging biological activities lies in understanding how its core mechanisms interconnect and reinforce one another across different therapeutic domains. antioxidant properties influence anti-inflammatory (through ROS reduction) and anticancer effects. Antimicrobial capabilities support wound healing, and selenium’s role in glucose metabolism underlies antidiabetic potential. This comprehensive investigation aims to understand these Se-NPs as versatile therapeutic agents capable of addressing multiple conditions through related biochemical pathways.

Therefore, considering all the above, the main objectives of the current study are the green production of Se-NPs by the marine actinobacterium *Streptomyces vinaceusdrappus* AMG31 isolated and identified by Ghareeb et al. [25] and the thorough characterization of these biogenically synthesized Se-NPs to assess their biomedical applications. Systematic assessment of their antioxidant potential through several different assays, investigating their hemocompatibility, wound healing, and anticancer activities. Assessment of their antidiabetic impact using enzyme inhibition assays, evaluation of antifungal and antibacterial potential, evaluation of antibiofilm activity against pathogenic microbes, and assessment of their anti-inflammatory activity.

## Materials and methods

### Biogenic Se-NPs synthesis using Streptomyces vinaceusdrappus AMG31

*Streptomyces vinaceusdrappus* AMG31, an actinobacterium isolated from Red Sea sediment near Marsa Allam city, Egypt, was used for the biogenic fabrication of Se-NPs. This strain was previously morphologically and genetically identified with the accession number (OR793047) [25]. The biosynthesis commenced by inoculating a single colony of the actinobacterium into 100 mL of Czapek Dox broth and incubating it at 30 ± 2 °C for 7 days with 150 rpm shaking. Post-incubation, the biomass was filtered using Whatman No.1, washed thrice with sterile distilled water, and 7 g was re-suspended in 100 mL distilled water (dH_2_O) and incubated again at 30 ± 2 °C for 24 h. The biomass filtrate obtained after centrifugation was used for Se-NPs fabrication by dissolving 85.5 mg Na₂O₃Se in 10 mL dH_2_O and mixing it with 90 mL of the biomass filtrate, creating a final ratio of 1:9 to achieve a 5 mM concentration. This mixture was stirred for an hour at 40 °C, pH adjusted to 8 using 1 N NaOH, and incubated overnight in the dark at 25 °C. The ruby red color formed, and the surface plasmon resonance (SPR) peak was detected between 200 and 700 nm wavelengths, using a control without sodium selenite, confirming the formation of Se-NPs. Finally, the Se-NPs were collected, triple-rinsed with distilled water, and oven-dried at 100 °C for 4 h to avoid the transformation of synthesized nanostructure to trigonal Se or SeO₂ [26].

### Characterization of Se-NPs

The Fourier transform infrared (FT-IR) spectroscopy technique, employing the Cary-660 model, was utilized to identify and characterize the vibrational modes associated with various chemical bonds and functional groups in the biosynthesized SeNP. A small quantity of 10 milligrams of Se-NPs was thoroughly mixed with KBr, and subsequently, this mixture was compressed into a thin, uniform, disk-shaped pellet. This pellet was then subjected to infrared radiation scanning within the wavenumber range of 400 to 4000 cm^−1^ [27]. The morphological characteristics, including shape and size, were examined using transmission electron microscopy (TEM, JEOL, Ltd-1010, Tokyo, Japan). Briefly, Se-NPs powder was suspended in highly pure Q-H_2_O under ultrasonication. Subsequently, a few drops of the suspension were added onto a TEM-carbon grid, which was then allowed to dry before being analyzed [28, 29]. The crystallographic structure of actinobacterial-mediated green synthesis of Se-NPs was evaluated using X-ray diffraction (XRD, PANalytical-X’Pert-Pro-MRD) with a CuKα electrode as the X-ray source (λ = 1.54 Aº). The analysis was conducted at a current of 30 mA and a voltage of 40 kV, within the 2θ range of 10°–80°. The elemental compositions of biosynthesized Se-NPs were assessed using EDX (JEOL, JSM6360LA, Japan) analysis.

The dynamic light scattering (DLS) technique, using a Nano-ZS instrument from Malvern Ltd., Malvern, UK, was employed to investigate the size distribution of the Se-NPs in the colloidal solution.

For DLS measurements, 0.5 mg mL^−1^ of Se-NPs were dispersed in Milli-Q H_2_O and sonicated for 15 min to ensure uniform dispersion. Measurements were performed at 25 °C with a scattering angle of 173° (backscatter detection) using a 633 nm He-Ne laser. Three independent measurements with 12–15 runs per measurement were conducted to ensure statistical reliability [30]. The synthesized Se-NPs were dispersed in a high-purity solvent (Milli-Q H_2_O) to prevent the appearance of shadows on the signal during scattering analysis. Additionally, the surface charge of the synthesized Se-NPs was assessed using a Zeta-sizer apparatus (Nano-ZS, Malvern, UK) at pH 7.0 using disposable folded capillary cells at an applied voltage of 150 V, with reported values representing the average of triplicate measurements [10].

### Biomedical applications

#### Antioxidant activity

##### 2,2-diphenyl-1-picrylhydrazyl (DPPH) Testing

The DPPH assay was used to determine the ability of Se-NPs to scavenge free radicals. A 0.1 mM of DPPH solutions in C_2_H_5_OH was formulated, and then 1 mL of this mixture was added to 3 mL of Se-NPs solutions ranging from 3.9 to 1000 µg mL^–1^. After this, the absorbance was recorded at the wavelength of 517 nm using a UV-Vis spectrophotometer (Milton Roy). As a reference standard compound, ascorbic acid was employed, the assay was performed in triplicate, and the scavenging percentage was calculated as follows: [31].

1 $$\:DPPH\:scavenging\: \%=\:\frac{Aa-Ab}{Aa}\:x\:100$$

Where Aa is the absorbance of the DPPH control reaction, and Ab is the absorbance in the presence of the Se-NPs or standard sample.

##### 2,2’-azino-bis(3-ethylbenzothiazoline-6-sulphonic acid) ABTS·^+^)radical scavenging activity

АBTS stock solution was dissolved in dH2O to a 7 mM concentration. The ABTS radical cation ABTS·^+^ was generated from ABTS stock solution by adding K_2_S_2_O_8_ at a concentration of 2.45mM and keeping the mixture at 22 °C in the dark for 12–16 h prior to use. The reaction mixture contained Se-NPs at 3.9 to 1000 µg mL^–1^, 0.07 mL of the Se-NPs, and 3 mL of the diluted ABTS·^+^ solution. Six minutes after the incubation, absorbance readings were taken using a spectrophotometer at 734 nm (M. A. Alharbi et al., 2023). The radical scavenging activity of ABTS was calculated as follows:

2 $$\:\text{A}\text{B}\text{T}\text{S} ^{\cdot+}\:\text{s}\text{c}\text{a}\text{v}\text{e}\text{n}\text{g}\text{i}\text{n}\text{g}\: \%=\:\frac{Control\:absorbance-Sample\:absorbance}{Control\:absorbance}x100$$

##### Total Antioxidant Capacity (TAC)

TAC of Se-NPs was evaluated by employing the Phosphomolybdenum method. A 0.5 mg mL^–1^ solution of Se-NPs was mixed together with a reagent mixture consisting of 0.6 M H_2_SO_4_, 28 mM NaH_2_PO_4_, and 4 mM ammonium molybdate. As a control, a solution without the Se-NPs was prepared, which only contained the reagent mixture. These mixtures were then held at 95 °C in an incubator for 150 min. When the mixtures were cooled to room temperature, absorbance measurements were recorded at 630 nm using a microtiter plate reader (Biotek ELX800; Biotek, Winooski, VT, USA) [32]. Results were presented as ascorbic acid equivalent (AAE) in µg/mg.

##### Ferric Reducing Antioxidant Power (FRAP)

A total of 40 µL of Se-NPs solution was dispensed into labelled Eppendorf tubes, then 50 µL of the solution was pipetted into the buffer containing 0.2 mol/L Na₂HPO₄_·_2 H₂O, 50 µL of 1% K₃[Fe(CN)₆], and 50 µL of 10% C₂HCl₃O₂. The mixture was centrifuged at 3000 rpm for 10 min. Then, 160 µL of the supernatant obtained from each Se-NPs tube was put into a 96-well microplate. After this, 30 µL of 1% FeCl_3_ solution was added. Absorbance measurements were taken at a wavelength of 630 nm with a microplate reader (Biotek ELX800; Biotek, Winooski, VT, USA) [33]. DMSO was employed as a negative control, and ascorbic acid (1 µg mL^–1^) served as a positive control. The results were reported in ascorbic acid equivalents (AAE in µg/mg), indicating the bioactive Se-NPs’ antioxidant capacity under different solvent polarities.

#### Wound healing

Human normal skin fibroblasts (HFB4, Cat. No. VACSERA-HFB4-005) were obtained from Biological Products and Vaccines (VACSERA), Cairo, Egypt, an accredited blood transport and cell culture centre, and seeded into a multi-well culture plate and left to grow to the confluence level of forming a single cell layer. A solution of Se-NPs at a conc. of 209.87 µg mL^–1^ was applied, and a scratching tool, specifically a yellow pipette tip tilted at about 30 degrees, produced a standard, straight scratch created to imitate a linear, uniform wound. This angled technique enabled dual imaging of the scratch on both sides at a 10x objective lens on the microscope [34].

#### Hemocompatibility assessment

Human red blood cells (RBCs, Cat. No. VACSERA-RBC-2023) were purchased from VACSERA, Cairo, Egypt, an accredited blood transport and cell culture centre. We confirm that the RBCs used in this research were commercially purchased. The ethical approve for blood and cell lines handling with number REC125/2025 was taken from Research Ethics Committee (REC), Faculty of Science, Suez Canal University. The collected RBCs were subjected to three washes with 150 mM NaCl, spun at 2500 RPM for 10 min, and subsequently resuspended in phosphate-buffered saline (pH 7.4) to prepare a 2% RBC suspension. The Se-NPs were evaluated at 25–1000 µg mL^–1^ concentration. Deionized water was used as the positive control (representing 100% hemolysis), while 0 µg mL^–1^ Se-NPs served as the negative control. Phosphate buffer saline was used as a blank for spectrophotometric measurements. Se-NPs serial dilutions, along with RBC solution, were combined to reach a final volume of 1 mL. The incubation lasted at 37 °C for 60 min. After the centrifugation at 2500 RPM for 15 min, the resulting supernatant was collected and measured at 546 nm [35]. All measurements were performed in triplicate to obtain mean ± S.D.

3 $$\:\text{H}\text{e}\text{m}\text{o}\text{l}\text{y}\text{s}\text{i}\text{s}\:\text{p}\text{e}\text{r}\text{c}\text{e}\text{n}\text{t}\text{a}\text{g}\text{e}\:\%=\:\frac{Ans.\:treatment-Abs.\:\:blank}{Abs.\:positive\:control}\:x\:100$$

#### Cytotoxicity

To assess the cytotoxic activity of Se-NPs using crystal violet viability assay, three different cell lines were investigated: the WI38 human fibroblast cell line (ATCC CCL-75), the Caco-2 human colon adenocarcinoma cell line (ATCC HTB-37), and the PANC-1 human pancreatic cancer cell line (ATCC CRL-1469). The selected cells were purchased from VACSERA, Cairo, Egypt, an accredited blood transport and cell culture centre. We confirm that the selected cell lines used in this research were commercially purchased and did not require ethical approval. Each cell type was separately cultured into a 96-well plate, with approximately 10³ cells per well, and rinsed in the growth medium. After an initial incubation period of 24 h, whereby cell attachment was observed, the cells were treated with different concentrations of Se-NPs (31.25–1000 µg mL^−1^) while the control cells received untreated control (medium only) and vehicle control (DMSO at highest concentration used). Each concentration was tested in triplicate wells. The plates were incubated for 24 h at 37 °C under 5% CO_2_ conditions in a humidified atmosphere [36]. After incubation, the growth medium was removed from each well, and wells were washed 2–3 times with PBS, and any remaining NPs were washed off using dH_2_O. Afterward, the wells were filled with a 1% crystal violet staining solution, the plates were then slightly shaken, and the plates were kept at room temperature for 10–15 min for cells to uptake the dye. After crystal violet staining, wells were washed 3–4 times with dH_2_O to remove unbound dye. The bound crystal violet was solubilized with 200 µL of 30% glacial acetic acid per well, followed by gentle shaking for 10 min. Finally, each well’s absorbance was read at 490 nm using a microplate ELISA reader [37, 38]. Microscopic images were captured for each concentration tested across all three cell lines to document morphological changes.

4 $$\:\text{C}\text{e}\text{l}\text{l}\:\text{v}\text{i}\text{a}\text{b}\text{i}\text{l}\text{i}\text{t}\text{y}\:\%=\:\frac{Abs.\:treatment}{Abs.\:control}x100$$

#### Antidiabetic

##### α-amylase inhibition

The ability of Se-NPs to inhibit the α-amylase enzyme was examined by using the 3.5 dinitro salicylic acid (DNS) method [39]. For this experiment, concentrations of Se-NPs ranging from 1.95 to 1000 µg mL^–1^ were tested, and acarbose was used as a comparison. A known α-amylase inhibitor (acarbose) was a positive control across the same concentration range. Absorption measurements were taken at the 540 nm wavelength using a UV-visible Biosystem 310 spectrophotometer. To determine the IC_50_ values that correspond to 50% inhibition where Se-NPs are acting on the increase of α-amylase activity, the graphical representation of the percentage of α-amylase inhibition and the corresponding Se-NPs concentrations was employed.

##### α-Glucosidase inhibition

The effect of Se-NPs on the α-glucosidase enzyme was assessed in a concentration range from 1.95 to 1000 µg mL^–1^. The activity of acarbose, which was employed as a positive control in the same tested concentration range, was compared with the Se-NPs enzyme inhibition activity. Subsequent absorbance measurements were taken by a Biosystem 310 Plus spectrophotometer at a wavelength of 405 nm. The IC_50_ values, which represent the concentration of Se-NPs required to inhibit 50% of the α-glucosidase, were determined using the regression equation plotted from the percentage of inhibition against a set of Se-NPs concentrations of between 1.95 and 1000 µg mL^–1^ [39].

#### Antimicrobial activity

The antibacterial activity of Se-NPs was evaluated using the agar well diffusion method against a broad spectrum of bacterial species obtained from the ATCC collection. This included Gram-positive strains (*Bacillus subtilis* ATCC 6633, *Staphylococcus aureus* ATCC 6538, and *Enterococcus faecalis* ATCC 29212) and Gram-negative strains (*Escherichia coli* ATCC 8739, *Klebsiella pneumoniae* ATCC 13883, *Pseudomonas aeruginosa* ATCC 90274, and *Salmonella typhi* ATCC 6539) on Mueller-Hinton agar media. Additionally, the antifungal activity was assessed against *Aspergillus niger* (AUMC 14260), *Mucor circinelloides* (AUMMC 11656), *Trichoderma harzianum* (AUMC 5408), *Penicillium glabrum* (OP694171) (AUMC15597), and *Candida albicans* (ATCC 10221) on sabouraud dextrose agar. Gentamicin was used as the control for bacterial inhibition screening, while fluconazole served as the antifungal control.

The inoculum suspension was prepared according to the standard broth dilution method, and the agar plates were inoculated within 15 min of preparation. The dried agar surface was streaked in three different directions to ensure even distribution of the inoculum. After allowing 15 min for the agar to dry completely, a sterile cork borer with a diameter of 6 mm was used under aseptic conditions to create wells in the agar. A solution of Se-NPs was prepared by dissolving it in DMSO at a concentration of 10 µg mL^–1^, and 100 µL of this solution was carefully pipetted into each well. The plates were then incubated for varying durations depending on the microorganism: 24 h for bacterial strains, 16–24 h for *Mucor circinelloides*, 24 h for *Aspergillus niger*, and 48 h for *Candida albicans*, *Trichoderma harzianum*, and *Penicillium glabrum*. After the respective incubation periods, the zones of inhibition were measured to the nearest whole millimetre at the point where significant growth reduction was observed [40]. Subsequently, the procedures defined by the Clinical and Laboratory Standards Institute (CLSI) approach were used to determine minimum inhibitory concentrations (MICs) and minimum bactericidal concentrations (MBCs) [40].

#### Antibiofilm

The Se-NPs’ effect on biofilm formation was evaluated using 96-well polystyrene plates. In brief, 300 µL of trypticase soy yeast broth (TSY) was inoculated with 10^6^ CFU/mL of bacteria and supplemented with sub-inhibitory levels of Se-NPs, representing 75%, 50%, and 25% of MBC. Following 48 h of incubation at 37 degrees centigrade, the biofilm that developed on the plates during the incubation period was stained with crystal violet for 15 min. The excess stain was then washed with sterile dH_2_O. The stained cells were then solubilized by adding 250 µL of 95% ethanol to each well. Incubation lasted for about 15 min, followed by absorbance measurement at 570 nm using a microplate reader, which indicated biofilm quantification [41].

5 $$\:\text{B}\text{i}\text{o}\text{f}\text{i}\text{l}\text{m}\:\text{i}\text{n}\text{h}\text{i}\text{b}\text{i}\text{t}\text{i}\text{o}\text{n}\:\% =1-\:\frac{A\:\left(sample\right)-A\left(blank\right)}{A\left(control\right)-A\left(blank\right)}x100$$

Where A(sample), A(blank), and A(control) represent the absorbance at 570 nm for the sample (containing the media, test organism, and 25%, 50%, or 75% MBC of Se-NPs), blank (media only), and control (media inoculated with organism only) wells, respectively.

####  Anti-inflammatory investigation

Se-NPs underwent evaluation for anti-inflammatory potential through COX-1 and COX-2 inhibition screening. The inhibitor assays were performed using cyclooxygenase-1 (COX-1, catalog number k548) and cyclooxygenase-2 (COX-2, catalog number k547) inhibitor assay kits provided by Biovision, USA. Se-NPs were prepared in DMSO and diluted from 1000 to 0.5 µg mL^–1^ in 1 mL of total volume [42, 43]. Celecoxib was used as a positive control for both assays. The COX-inhibition percentages were measured as follows:

6 $$Cox-inhibition \% =1- \frac{Absorpance\,of\,treatement}{Absorpance\,of\,control} \times 100$$

### Statistical analysis

SPSS (version 22, USA) was used for data analysis. The Shapiro-Wilk, Equal Variance, and One-Way Analysis of Variance (ANOVA) tests were performed. Following the ANOVA, a post hoc test was conducted. Experiments were done in triplicate and results expressed as mean ± SD, (*n* = 3, *p* < 0.05)

## Results and discussion

### Characterization of the biosynthesized Se-NPs

**Fig. 1:**
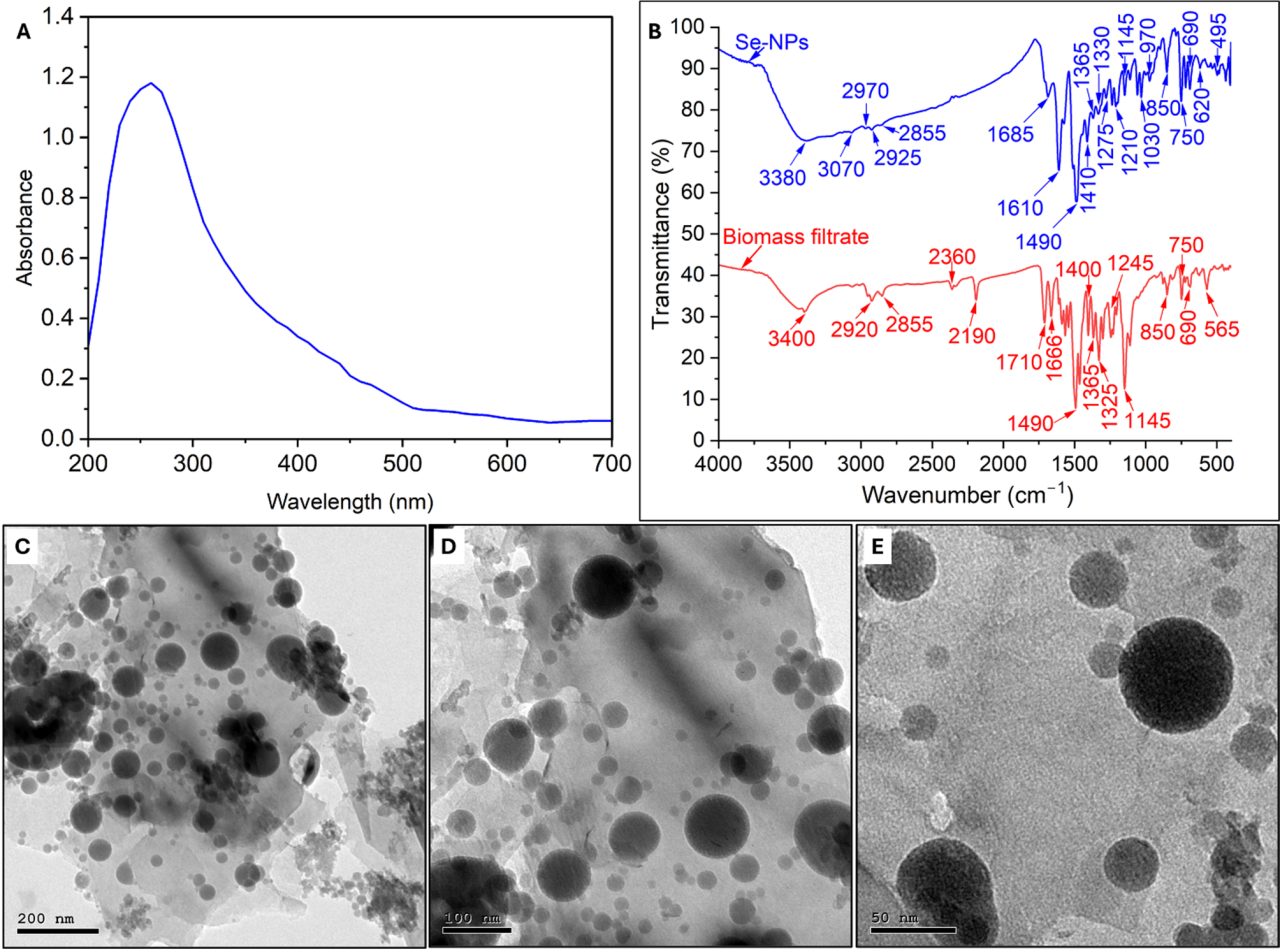
Characterization of actinobacterial-driven biogenic Se-NPs. (**A**) UV-vis spectroscopy shows the maximum SPR, (**B**) FTIR shows different functional biomolecules, and (**C**–**E**) TEM images at various magnification powers show a spherical shape

Recently, the biosynthesis of nano-selenium structures has received high attention from researchers because they are considered necessary micronutrients for living organisms. Therefore, the synthesis of Se-NPs has increased in the last decades, especially by green approaches. Herein, *Streptomyces vinaceusdrappus* strain AMG31 was utilized to fabricate Se-NPs and to assess their biomedical applications. The formation of ruby color after interaction of Na₂O₃Se with the actinobacterial biomass filtrate confirmed the successful fabrication of Se-NPs. The intensity of this ruby color was monitored using UV-vis spectroscopy to measure the surface plasmon resonance (SPR). Figure 1A showed the maximum SPR for actinobacterial synthesized Se-NPs was 260 nm, which referred to the successful formation of small sizes and spherical shape [44]. Whereas the UV of biomass filtrate showing λ_max_ at wavelength of 290 nm (Fig. S1, see supplementary data) which refers to the presence of aromatic compounds such as phenolic compounds (tannins and flavonoids), aromatic amino acids (tryptophane and tyrosine), and extracellular metabolites (such as enzymes, pigments, antibiotics) [3]. Recently, the maximum SPR of Se-NPs fabricated by different fungal strains appeared in the range of 260–280 nm [45]. The bacterial strain, *Ralstonia eutropha*, was utilized to fabricate Se-NPs with maximum SPR at 270 nm [46]. The Se-NPs reproducibility synthesis is mainly some parameter-dependents such as biomass filtrate production under optimum conditions for actinobacterial growing, pH, metal precursor concentration, contact times, and temperature. In our study, the S. vinaceusdrappus was grown under the same conditions at each batch (Czapek Dox broth at 30 ± 2 °C for 7 days with 150 rpm shaking) to confirm the production of same secondary metabolites that used as reducing agent. Moreover, the same concentration of Na₂O₃Se was mixing with biomass filtrate under pH value of 8 and stirring with 40 °C for 60 min followed by dark incubation for 24 h to ensure consistent batch-to-batch reproducibility. Under these optimum conditions, ensuring scalability and production of maximum yield at each batch.

FT-IR for actinomycetes biomass filtrate and biosynthesized Se-NPs exhibits different functional peaks at varied wavenumbers (Fig. 1B). As shown, the broad and strong peak at 3400 cm^–1^, shifted to 3380 cm^–1^ after nano-selenium fabrication, refers to stretching O-H of alcohol or N-H of amines [47]. The weak peaks at the wavenumbers in the 3100–2800 cm^–1^ range correspond to the stretching O-H group of phenol, alcohol, or water [48]. The strong peak at 1666 cm^–1^, deconvoluted into two peaks at 1685 and 1610 cm^–1^ upon Se formation, is related to the bending N-H of secondary amines, whereas the peaks at 1490 and 1400 cm^–1^ (shifted to1410 cm^–1^ in Se-NPs) correspond to the C = C or aromatic compounds [49]. The peaks in the ranges of 1365 to 1330 cm^–1^ in Se-NPs and biomass filtrate charts are related to the bending OH of phenol, whereas the peak at 1275 cm^–1^ signifies the C-N of aromatic amines, or S = O of sulfonates, or C-O of carboxylic acid, ethers, or ester [50]. The presence of different peaks in the ranges of 1000–1200 cm^–1^ indicates the presence of polysaccharides and C-O-C of sugars [51]. The peaks in the ranges of 400–600 cm^–1^ signify the alkaline halides (C-Se) [52]. The presence of these different functional groups indicates the efficacy of actinobacterial metabolites, such as proteins, polysaccharides, amines, and carbohydrates, in the reduction of Na₂O₃Se to form Se-NPs, followed by capping and improving their stability.

The detection of the sizes and shapes of the biogenic Se-NPs was achieved by electron microscopy analysis. The obtained images (Fig. 1C–E) revealed that the biosynthesized Se-NPs exhibited a spherical morphology with sizes ranging from 20 to 80 nm. Recently, *Streptomyces parvulus* was utilized to formation of semispherical Se-NPs with a size of 94 nm [53]. Also, *Streptomyces minutiscleroticus* was used to produce spherical Se-NPs with sizes of 100–250 nm, and the authors investigated antioxidant, antibiofilm, anticancer, wound healing, and anti-viral activities on it [54]. The activity of Se-NPs, especially in biomedical applications, mainly depends on their size, shape, agglomeration, stability, and surface charges. The activity was enhanced with smaller sizes compared to bigger ones. For instance, garlic-mediated biosynthesis of Se-NPs exhibited promising antimicrobial activity with sizes of 21–40 nm compared to their activity with sizes of 41–50 nm [55].

**Fig. 2:**
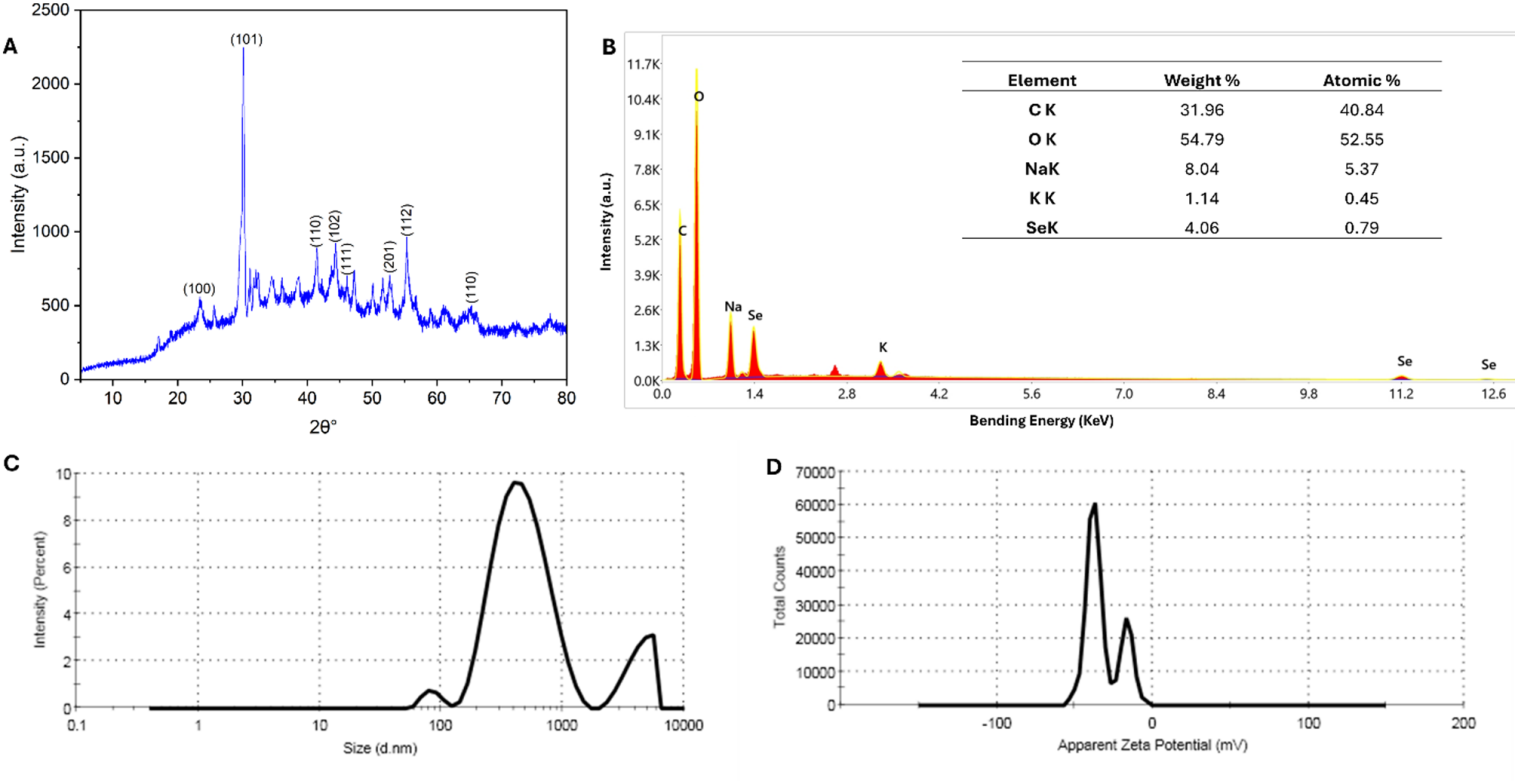
XRD analysis showing the crystalline nature of biogenic Se-NPs (**A**), elemental compositions of biogenic Se-NPs by EDX (**B**), size distribution in the colloidal by DLS (**C**), and stability detection using zeta-potential analysis (**D**)

The crystallinity of actinobacterial-synthesized Se-NPs was assessed using XRD. Figure 2A showed that the sample containing Bragg’s diffraction peaks of (100), (101), (110), (102), (111), (201), (112), and (202) corresponding to 2θ° of 23.4°, 30.13°, 41.38°, 44.32°, 46.14°, 52.73°, 55.3°, and 65.7° respectively. The obtained diffraction confirmed that the biosynthesized Se-NPs were crystalline in nature according to the standard file of JCPDS No. 06–362 [56]. Recently, the biogenic Se-NPs using *Penicillium crustosum* exhibit diffraction peaks of (100), (101), (110), (102), (112), (202), and (210) at 2θ° 23.2°, 29.6°, 40.3°, 43.5°, 50.1, 55.5°, and 66.2°, respectively [57]. Singh and coauthors reported that the presence of peaks (100), (101), and (102) at 23°, 30°, and 43° values of 2θ confirmed the efficacy of microbial metabolites to reduce Na₂O₃Se and form crystallographic Se-NPs structure [58]. The presence of extra peaks in the XRD chart could be attributed to the scattering of actinobacterial capping agents [59].

The elemental compositions of actinobacterial synthesized Se-NPs were detected using EDX analysis (Fig. 2B). The EDX chart shows the presence of Se ions in addition to C, O, Na, and K ions with varied weight and atomic percentages. The presence of peaks at 1.4, 22.1, and 12.6 KeV bending energies confirmed the formation of Se-nanostructure [60]. The presence of other peaks (matched with the XRD chart) could originate from capping biomolecules such as proteins, enzymes, and carbohydrates [61]. The high weight and atomic% of O due to oxidation or the presence of oxygen-containing organic coatings [62]. The obtained findings were compatible with different published investigations that confirmed the presence of other peaks in EDX chart with Se and returned it to capping bioactive molecules [63].

The hydrodynamic sizes of biogenic Se-NPs in the liquid were determined by DLS (Fig. 2C). As shown, there are three peaks represented the sizes of 493.5 nm (for intensity 84.4%), 431 nm (for 13.2%) and 84.9 nm (for 2.3%) with an average hydrodynamic size of 460.1 nm. Similarly, the DLS of Se-NPs formed by exopolysaccharide secreted from *Bacillus* sp. showed hydrodynamic sizes in the ranges of 200–300 nm with an average of 209 nm [64]. Also, the sizes of Se-NPs formed by bacterial strain *Zooglea ramigera* ranged from 78 nm to 210 nm with an average of 152 nm as detected by DLS analysis [65]. Usually, the sizes obtained by DLS are higher than TEM sizes due to the DLS measuring the sizes in the hydrated state (hydrodynamic size), whereas TEM measures the particle at solid state [45]. Also, the DLS is affected by the capping agent in the liquid solution. Moreover, during sample preparation for TEM analysis involves drying and high-vacuum conditions, which leads to weak agglomerates. Whereas, DLS reflects the colloidal state in suspension, where leads to mild to moderate agglomeration due to electrostatic or van der Waals interactions, even if the particles are well-dispersed under TEM. The non-well distribution or aggregation of the NPs in the liquid affected DLS analysis and gave large sizes [66]. The homogeneity percentages of synthesized Se-NPs in the liquid solution can be detected by measuring the polydispersity index (PDI) during DLS analysis. PDI has a range from 0 to 1; the heterogenous increased when the PDI value was close or equal to 1, whereas the homogeneity increased at a PDI value less than 0.4 and decreased at a value greater than 0.4 to 1 [67]. Here, the PDI value of synthesized Se-NPs was 0.384, indicating the high homogeneity and stability of NPs in the colloidal solution. Similarly, the PDI of Se-NPs produced by *Z. ramigera* bacterial strain was 0.438 [65], which indicates (as the authors mentioned) high particle stability.

The zeta-sizer of synthesized Se-nanostructure showed two peaks with a zeta potential value of −37.9 mV for the first peak (high peak) and −16.9 mV for the second peak (low peak) (Fig. 2D). The presence of one charge (-ve) on the Se-NPs surface indicates the electrostatic repulsion between particles and each other’s, leading to aggregation stopping, and hence high stability [68]. Some authors reported that the capping agents, such as flavonoids, terpenoids, polysaccharides, and alkaloids, secreted by biological entities and used for reducing the metal and production of the nanoscale structure have a role in improving stability via adding the -ve charge to NPs surfaces and hence enhanced electrostatic repulsion between particles [69].

### Biomedical applications

#### Antioxidant assessment of Se-NPs

All four assays, including DPPH, ABTS^+^, TAC, and FRAP, showed that Se-NPs possessed concentration-dependent antioxidant activity with promising antioxidant potential. In the measurements done with DPPH and ABTS, Se-NPs increased their radical scavenging ability with increasing concentration, which also meant that a greater degree of inhibition was achieved. In particular, at the highest concentration range, 1000 µg mL^–1^ of Se-NPs, the results were DPPH of 86.7% scavenging and ABTS antioxidant activity of 84.6%. At the lowest tested concentration of 1.95 µg mL^–1^, Se-NPs exhibited a 23.9% DPPH scavenging activity. As concentration increased, the scavenging percentage also increased. At 7.8 µg mL^–1^, the scavenging percentage was 36.7%, while at 15.6 µg mL^–1^, it reached 45.5%. A notable increase was observed at 31.25 µg mL^–1^, with a 52.9% scavenging activity. The scavenging percentage continued to rise, reaching 59.4% at 62.5 µg mL^–1^, 66.4% at 125 µg mL^–1^, and 72.9% at 250 µg mL^–1^. Its IC_50_ value was 26.06 µg mL^–1^ compared to ascorbic acid (2.54 µg mL^–1^).

**Fig. 3:**
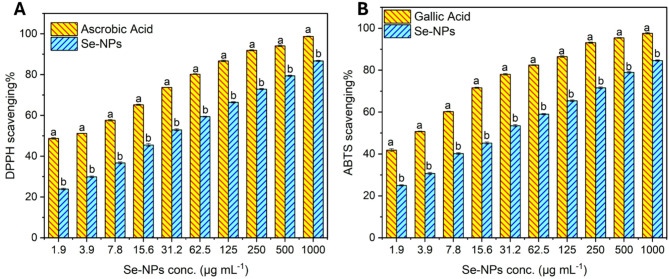
Antioxidant activity of actinobacterial-mediated biosynthesis of Se-NPs. (**A**) DPPH scavenging activity was compared to a positive control (ascorbic acid), and (**B**) ABTS scavenging activity of Se-NPs was compared to gallic acid as a positive control. Different letters (**a** and **b**) on the bars at the same concentration indicate the results are significantly different (Mean ± SD, *n* = 3, *P* ≤ 0.05)

At a concentration of 500 µg mL^–1^, Se-NPs exhibited a 79.4% DPPH scavenging ability, which further increased to 86.7% at the maximum tested concentration of 1000 µg mL^–1^ (Fig. 3A).

A similar pattern was demonstrated by Se-NPs in ABTS·^**+**^ assay, as the scavenging activity increased with an elevation in the concentration. Where at its lowest concentration of 1.9 µg mL^–1^, the ABTS scavenging percentage was 25.0%. This percentage increased to 30.7% at 3.9 µg mL^–1^, 40.2% at 7.8 µg mL^–1^, and 45.2% at 15.6 µg mL^–1^. A noticeable increase was observed at 31.2 µg mL^–1^, with a 53.5% scavenging activity. The IC_50_ value for Se-NPs was determined to be 25.17 µg mL^–1^ compared to 2.54 µg mL^–1^ of gallic acid. The scavenging percentage continued to rise, reaching 59.0% at 62.5 µg mL^–1^, 65.4% at 125 µg mL^–1^, and 71.6% at 250 µg mL^–1^. At a concentration of 500 µg mL^–1^, Se-NPs exhibited a 79.0% ABTS scavenging ability, which further increased to 84.6% at the maximum tested concentration of 1000 µg mL^–1^ (Fig. 3B).

The TAC and FRAP values provided an overall assessment of the antioxidant capacity of Se-NPs, expressed in terms of ascorbic acid equivalents (AAE). For Se-NPs, the TAC value was reported as 965.9 ± 4.9 µg/mg. This value represents the concentration of ascorbic acid that would exhibit an antioxidant capacity equivalent to 1 mg of Se-NPs. Meanwhile, the Ferric FRAP assay evaluates the ability of an antioxidant to reduce Fe^3+^ to Fe^2+^. For Se-NPs, the FRAP value was reported as 727.7 ± 7.6 µg/mg. This value indicates the concentration of ascorbic acid that would exhibit a ferric-reducing ability equivalent to 1 mg of Se-NPs (Table 1).

**Table 1:** Total antioxidant capacity (TAC) and ferric reducing antioxidant power (FRAP) tests analysis of Se-NPs

Se-NPs (AAE)µg/mg	TAC (equivalent (AAE) µg/mg) Mean ± SD	FRAP (equivalent (AAE) µg/mg) Mean ± SD
	965.9 ± 4.9	727.7 ± 7.6

Data expressed as ascorbic acid equivalent (AAE), Mean ± SD, n=3

Following our findings, a phytosynsized Se-NPs with an average size of 80 nm from *Nyctanthes arbortristis* L exhibited potent antioxidant activity in DPPH and H_2_O_2_ assays [70]. Another spherical Se-NPs sized 80 nm derived from *Diospyros montana* exhibited robust antioxidant activity, with IC_50_ of 24.7 ± 0.6 µg mL^–1^ and EC_50_ of 46.3 ± 0.2 µg mL^–1^ in DPPH and reducing power assays [71]. *Nocardia concave* was utilized to fabricate Se-NPs that was spherical and showed antioxidant capacity in DPPH and ABTS assays with IC_50s_ = 31.4 µg mL^–1^ and 35.7 µg mL^–1^, respectively [72]. Similarly, cyanobacterium *Anabaena* was employed to biofabricate Se-NPs that was spherical and sized 50 nm that demonstrated high antioxidant activity at 50 µg mL^–1^ in the DPPH assay [73].

ROS and reactive nitrogen species (RNS), referred to as free radicals, are produced during normal metabolic functions and have an essential role in the physiological processes of a cell. Conversely, when present in excessive amounts, these free radicals harm essential cellular components such as proteins, nucleic acids, and membranes [74]. It is worth mentioning that the Se-NPs can exhibit some antioxidant activity by lowering these oxidant species by donating electrons and converting them into non-reactive species. Furthermore, Se-NPs enhance and stimulate the production of essential antioxidant enzymes, including superoxide dismutase (SOD), and catalase (CAT) which scavenges H_2_O_2_ together with lipid and phospholipid hydroperoxides and converts them to water and alcohol [15]. Also, it has been reported that Se-NPs functioning as Glutathione peroxidase (GPx) mimetics that decompose peroxides via glutathione-mediated reactions, while concurrently stimulating Nrf2 transcription factors to boost production of vital protective enzymes as SOD, CAT, and heme oxygenase-1 [75].

#### Scratch wound healing assay

The scratch wound healing assay results indicated that Se-NPs at 209.87 µg mL^–1^ promoted enhanced wound healing compared to the control cells. Notably, the Se-NPs-treated cells exhibited a higher migration rate (10.4 μm h^–1^) than the control cells (10.4 μm h^–1^), suggesting an accelerated wound closure process. Furthermore, the percentage of wound closure was markedly higher in the Se-NPs group (73.6%) compared to the control group (62.6%), corroborating the wound-healing effect of Se-NPs. Moreover, the area difference percentage, which quantifies the change in the wound area in µm², was also considerably higher for the Se-NPs-treated cells (445876.7 μm²) than the control cells (379461.5 μm²), corresponding to area difference percentages of 73.6% and 62.6%, respectively further reinforcing the efficacy of Se-NPs in promoting wound healing (Table 2)(Fig. S2, see supplementary data).

**Table 2:** In vitro scratch assay wound healing of Se-NPs over 48 h

Treatment	at 0 h		at 48 h		RM µm h^–1^	Wound closure %	Area difference µm^2^
	Width µm	Area µm²	Width Μm	Area µm²			
Control cells	794.3	605793.9	296.7	226332.4	10.4	62.6	379461.5
Se-NPs	794.3	605793.9	209.7	159917.2	12.2	73.6	445876.7

Se-NPs offer dual protection in wound microenvironments through their powerful anti-inflammatory and antioxidant effects. They reduce inflammation by shifting macrophages from pro-inflammatory to anti-inflammatory states, speeding healing [76]. Also, their strong antioxidant properties combat oxidative stress in wounds, supporting essential cellular healing functions [77]. Furthermore, Coating Se-NPs with red blood cell membranes improves their stability and helps them evade immune detection, making them more effective against infected wounds [78]. This enhanced efficacy is evident in a study where fungal-derived Se-NPs significantly reduced *Staphylococcus aureus* infections, resulting in smaller wound areas and faster healing [79].

Se-NPs promote tissue regeneration by stimulating fibroblast proliferation and collagen synthesis, key elements in wound healing [80] Their effectiveness increases when combined with other treatments, as seen when paired with platelet-rich plasma (PRP), where they work synergistically with PRP’s growth factors to speed healing significantly [81]. Similarly, when integrated into nitric oxide-generating gels, Se-NPs enhance both collagen deposition and epithelialization, where wounds are healing [82].

#### Hemocompatibility examination

The hemolytic activity of Se-NPs was assessed against a positive control (complete hemolysis by deionized H_2_O, representing 100% hemolysis). Negative control (isotonic solution), where concentrations from 1000 to 25 µg mL^–1^ exhibited minimal hemolytic effects: 1000 µg mL^–1^ showed 1.8% hemolysis, 800 µg mL^–1^ causing 0.5% hemolysis, 600, 400, and 200 µg mL^–1^ causing 0.8%, 0.4%, and 0.2% hemolysis respectively.

On the other hand, the concentrations from 100 to 25 µg mL^–1^ maintained a consistent 0.1% hemolysis (Table 3). When compared to the complete hemolysis control (absorbance 1.211), these values demonstrated negligible membrane disruption (Fig. S3, see supplementary data), confirming the safety profile of Se-NPs towards red blood cells even at high concentrations.

**Table 3:** Quantitative hemolytic assessment: Se-NPs, complete hemolysis, and isotonic controls

		Absorbance Mean ± SD	Hemolysis (%)
Complete hemolysis (+ ve control)		1.211 ± 0.012	100
Isotonic solution (-ve control)		0.001 ± 0.001	0
Se-NPs Conc. (µg mL^–1^)	1000	0.088 ± 0.003	1.8
	800	0.049 ± 0.006	0.5
	600	0.037 ± 0.005	0.8
	400	0.019 ± 0.002	0.4
	200	0.009 ± 0.001	0.2
	100	0.003 ± 0.002	0.1
	50	0.002 ± 0.001	0.1
	25	0.002 ± 0.001	0.1

Hemolytic assays are crucial for assessing how Se-NPs interact with red blood cells, with research showing that specific biologically produced Se-NPs demonstrate minimal hemolytic activity, indicating favourable blood compatibility [83]. As evidence of this pattern, chitosan-stabilized Se-NPs caused only 7.2% hemolysis, substantially below toxic control levels, suggesting their suitability for safe medical applications [84].

Assessing blood compatibility for biogenic Se-NPs necessitates thoroughly examining multiple crucial factors to confirm their safety profile and effectiveness in medical safety and function. Manufacturing techniques, dimensional measurements, structural formations, and RBCs reactivity are central factors when determining compatibility between selenium-based nanomaterials and blood components. Phytogenic Se-NPs and microbial-synthesized Se-NPs exhibited distinct biological functionality and toxicity signatures compared to chemically manufactured versions [85].

Notably, environmentally sustainable fabrication using *Hybanthus enneaspermus* extracts [86] or *Bacillus halotolerans* cultures [87] has yielded remarkably compatible selenium nanostructures with minimal harmful effects. Dimensional and structural characteristics of Se-NPs play essential roles in their functionality; particularly, minute small particles measuring 5–50 nm typically demonstrate superior cell-level engagement and may significantly alter blood compatibility outcomes [88]. Also, Round-formed Se-NPs consistently show improved tissue integration and lower rates of blood cell rupture [89].

#### Anticancer evaluation of Se-NPs

**Fig. 4:**
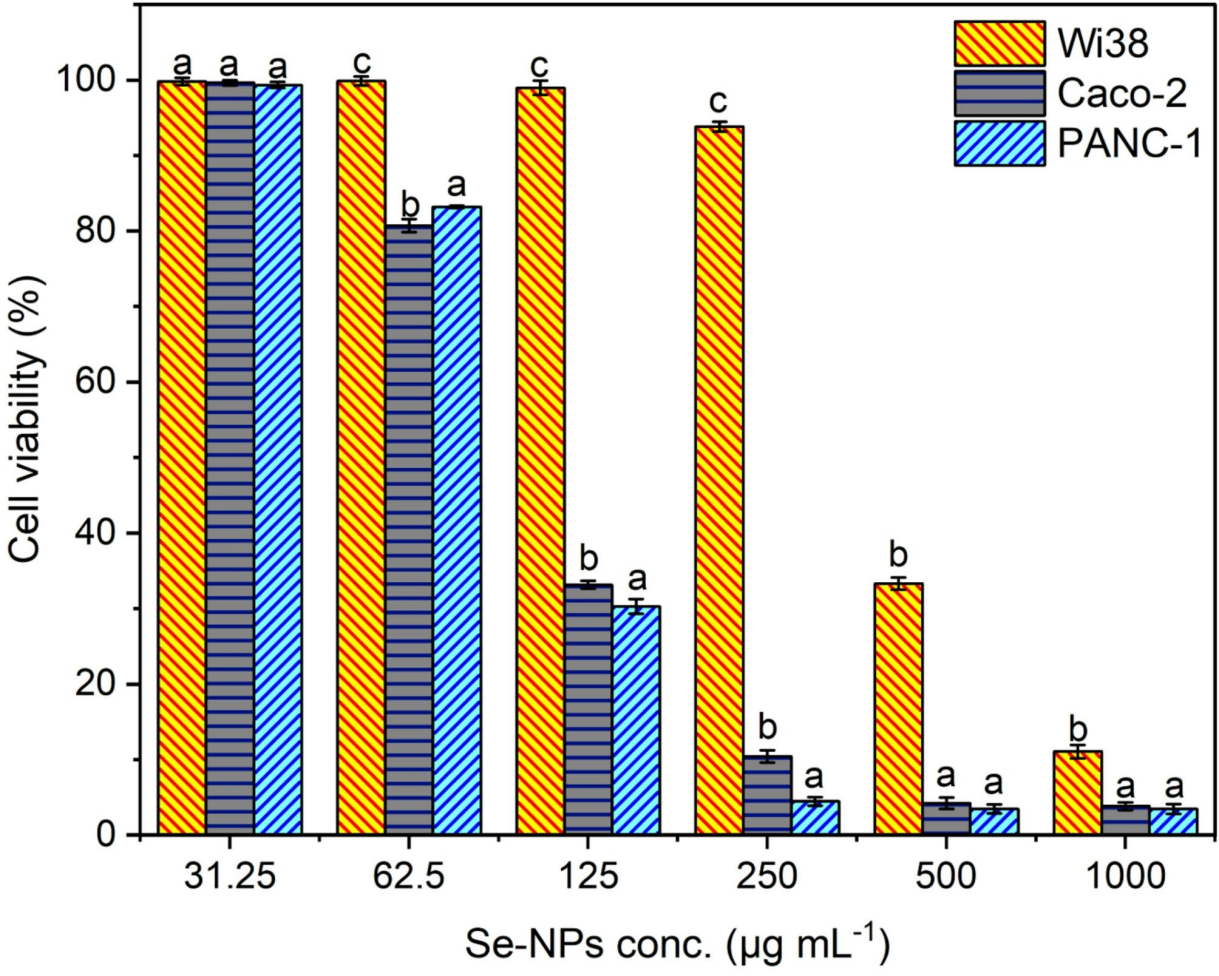
Cell viability of Wi38, Caco-2, and PANC-1 cells treated with biogenic Se-NPs (31.2–1000 µg mL^–1^). Different letters (**a**, **b**, and **c**) on the bars at the same concentration indicate the results are significantly different (*n* = 3, *P* ≤ 0.05)

The evaluation of biocompatibility of Se-NPs against normal WI-38 cells showed minimal or no cytotoxicity for all the concentrations studied with the exception of concentrations 1000 and 500 µg mL^–1^, where the percentage of cell viability dropped to 11.1 and 33.3%, respectively (Fig. 4). It is observed that as the concentration decreased, the cytotoxicity was reduced, while the cell viability was significantly high, remaining at 93.8% for 250 µg mL^–1^ and above 98% for concentrations ranging from 125 µg mL^–1^ to 31.25 µg mL^–1^, likely with minor or no morphological changes as in the case of control cells (Fig. S4, See supplementary data). Interestingly, the IC_50_ value was found to be noteworthy, 419.7 ± 5.2 µg mL^–1^.

For the anticancer activity against the Caco-2 cell line, Se-NPs also demonstrated concentration dependence, where increased concentrations were more cytotoxic and accompanied by visible morphological changes. Particularly, the most profound cytotoxicity occurred at the highest tested concentration of 1000 µg mL^–1^, where the cell viability decreased to only 3.8% (Fig. 4), signifying cytotoxic effects, evidenced by noticeable cytomorphological changes. At this concentration, cells rounded up and completely lost attachment to the surface, which are signs of cell death or apoptosis caused by the potent cytotoxic impact of Se-NPs during treatment (Fig. S5, See supplementary data). A similar concentration-dependent trend was observed at the concentration of 500 µg mL^–1^, where cell viability deteriorated to as low as 4.2% (Fig. 4), suggesting considerable cytotoxicity was present along with striking morphological distortions, possibly resembling the cellular deformations observed at the highest concentration (Fig. S5, See supplementary data). As the concentration dropped to 250 µg mL^–1^, the cell viability increased to 10.5% (Fig. 4). However, this still reflected severe cytotoxicity, and these are likely to be accompanied by changes in the morphological features like cell shrinkage or atypical cellular shapes, pointing to the fact that Se-NPs still interfered with the cell structure. Even at 33.2% viability with a 125 µg mL^–1^ concentration, the morphological alteration was rather expected but less severe than that at higher concentrations. Remarkably, it is interesting to note that at lower concentrations of 62.5 and 31.25 µg µg mL^–1^, the observed cell viability increased significantly to 80.7% and 99.9%, respectively (Fig. 4), evidencing very low levels of cytotoxicity and slight morphological changes as compared to the untreated control cells (Fig. S5, See supplementary data). Furthermore, the determined IC_50_ of 102.5 ± 2.1 µg mL^–1^ complemented this concentration-dependent cytotoxic effect, with cell death and disruption of cell morphology being significant at concentrations above this value and less morphometric abnormalities with some cytotoxicity at concentrations below.

Our quantitative analysis of the PANC-1 pancreatic cancer cell line showed a concentration-cytotoxic inhibitory effect of Se-NPs with an IC_50_ value of 100.4 ± 1.9 µg mL^–1^. Cells treated with Se-NPs at 250, 500, and 1000 µg mL^–1^ showed a severe cytotoxic effect as the viability percent ranged between 3.5% and 4.5%. In contrast, lower 125 and 62.5 µg mL^–1^ concentration had a viability of 69.2% and 16.8%, respectively (Fig. 4).

The notable increase in cell viability at lower Se-NPs concentration also points toward the potential of minimal cytotoxicity and changes in cell morphologies since the untreated PANC-1 cells displayed seemingly epithelial-like morphology, which is characterized by a more or less fair cobblestone appearance with well-defined cell borders (Fig. S6, see supplementary data).

Microbial and photosynthesized Se-NPs, with a size range of 79 to 500 nm, have been reported to be promising candidates for malignancies treatment [90]. For example, spherical Se-NPs sized 22 nm bioformed from *Portulaca oleracea* have been reported to have anticancer activity against HepG2 with IC_50_ = 70 µg mL^–1^ [69]. Another mycofabricated spherical Se-NPs from *Penicillium verhagenii* was documented to have anticancer activity against MCF7 and PC3 cell lines [45]. Similarly, spherical Se-NPs derived from *P. crustosum* inhibited the cancerous cells of T47D and HepG2 cells lines in vitro [57]. Also, spherical Se-NPs with an average size of 48.9 nm bioformed from *P*. *corylophilum* were documented to have an IC_50_ = 104.3 ppm against Caco-2 cell lines, while retaining biocompatibility against normal WI38 with IC_50_ = 171.8 ppm [91].

Se-NPs have been proven to be effective against various cancers, including breast, prostate, and other cancers. In breast tumours, they demonstrated anticancer action by decreasing levels of certain pro-inflammatory cytokines such as IL-17, IL-2, IL-12, IFN-γ, and TNF-α, and improved delayed-type hypersensitivity and natural killer cells activity, which resulted in reduced tumor size and prolonged the life span of a mouse model of breast cancer [75, 92]. In prostate cancer PC-3 cells, the ameliorative effect of Se-NPs was demonstrated by the upregulation of necroptosis-related factors IRF1 and TNF, decreased levels of prostate-specific antigen (PSA) and androgen receptor (AR), and an enhanced ROS-dependent necroptosis in PC-3 cells [93]. Additionally, Se-NPs inhibited the tumor cells’ angiogenic signaling pathways, which in turn inhibited their proliferation and growth. All these damaging cellular events, which were preceded by DNA damage sequentially, ended up in cell cycle arrest and were eventually fatal to the cells [92].

Cancer cells typically maintain an acidic environment within their cytoplasm and redox imbalances. Such an internal environment assists the pre-oxidative modification of Se-NPs, leading to an upsurge in forming free radicals. This causes the disturbance of the mitochondrial membrane and the outflow of its proteins, and simultaneously, the endoplasmic reticulum stress is triggered as well [94]. Such leakage causes the efflux of different proteins and initiates apoptosis through the activation of caspases, proteases essential for apoptosis. Such cellular stress coordinates the triggering of several molecular signalling pathways, including MAPK/Erk, PI3K/Akt/mTOR, NFκB, Wnt/β-catenin, and apoptosis pathways [95, 96]. The NFκB pathway promotes the inflammatory process, which in turn causes cellular oxidative stress and disrupts homeostasis. The PI3K/Akt/mTOR, MAPK/Erk, VEGF, and Wnt/β-catenin signalling cascades are vital for oncogenic signalling [97], and their modulation by Se-NPs inhibits cellular proliferation and also antagonizes the growth promoting signalling within the tumour cell microenvironment.

#### Antidiabetic evaluation of Se-NPs

**Fig. 5:**
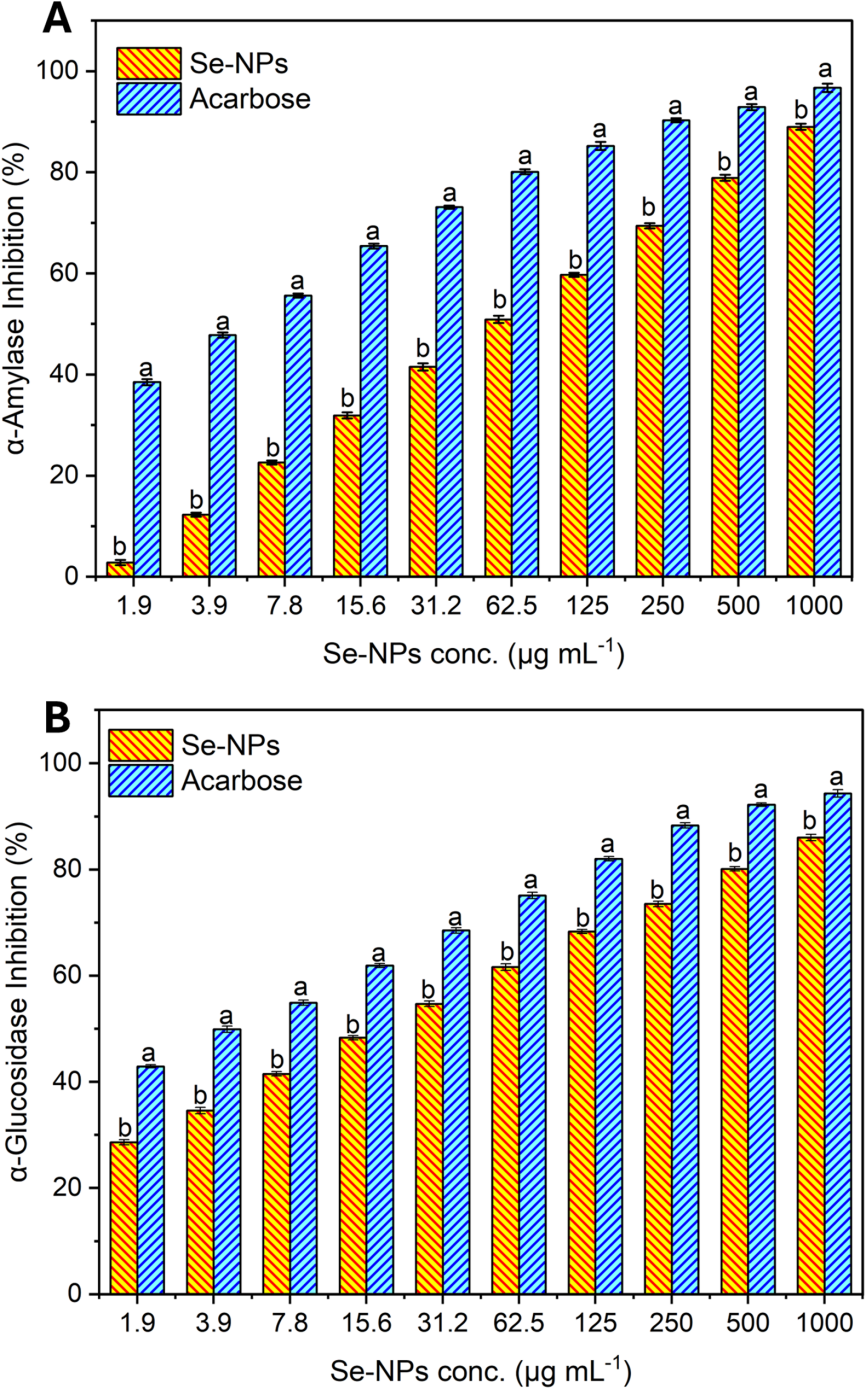
In vitro comparative inhibition % of α-amylase (**A**) and α-glucosidase (**B**) enzymes by Se-NPs vs. Acarbose (positive control) at concentrations of 1.9 to 1000 μg mL^-1^. Different letters (**a** and **b**) on the bars at the same concentration indicate the results are significantly different (*n* = 3, P ≤ 0.05)

The antidiabetic activity of biogenic Se-NPs was evaluated by its efficacy to inhibit α-amylase and α-glucosidase enzymes. Our study also revealed that the activity of α-amylase is inhibited with an increasing concentration of Se-NPs. The inhibition percentages were relatively low at lower concentrations of 1.9 and 3.9 µg mL^–1^ of Se-NPs’s, the inhibition percentages were 2.8% and 12.3%, respectively. Nevertheless, with higher concentrations of Se-NPs, the inhibition percentages were higher. For instance, at 7.8 µg mL^–1^, the inhibition percentage reached 22.6%, progressively increasing to 31.9%, 41.5%, and 50.9% at 15.6, 31.2, and 62.5 µg mL^-1^ concentrations, respectively compared to 65%, 73% and 80% enzyme inhibition by acarobose at same tested concentrations (Fig. 5). The observed increase in the inhibition percentages with increased levels of Se-NPs confirms the concentration dependent inhibitory effect. Where highest inhibition of 89.0% was observed at a Se-NPs concentration of 1000 µg mL^–1^ with an IC_50_ of 59.8 µg mL^–1^ compared to 4.03 µg mL^–1^ by acarbose.

An analogous concentration-dependent activity of inhibition of α-glucosidase enzymes was observed. On lower dose levels of 1.9 and 3.9 µg mL^–1^, inhibition percentages were 28.6 and 34.6%, respectively, which indicate moderate inhibition. As the concentration elevated, the inhibitory effect augmented proportionally, with inhibition percentages reaching 41.5, 48.3, and 54.7% at concentrations of 7.8, 15.6, and 31.2 µg mL^–1^, respectively compared to 41.5%, 48.3%, and 54.7% enzyme inhibition by the control acarbose at the same tested concentrations. Furthermore, the inhibition continued to rise substantially, reaching 61.6, 68.3, 73.5, and 80.1% at 62.5, 125, 250, and 500 µg mL^–1^ concentrations, respectively (Fig. 5). Se-NPs showed a maximum inhibition of 86.0% at the maximum concentration of 1000 µg mL^–1^, while the concentration at which 50% inhibition was observed was 19.26 µg mL^–1^ vs. 3.92 µg mL^–1^ by acarbose.

Following our results, spherical Se-NPs with an average size of 250 nm derived from *Moringa oleifera* demonstrated concentration dependent inhibitory effects on both enzymes α-amylase and α-glucosidase, with inhibition ratios of 44.5 and 19%, respectively [98]. Similarly, another biofabricated Se-NPs by *Mimosa pudica* exhibited a dose proportional suppression of β-glucosidase and α-amylase in vitro [99]. In another study, Se-NPs synthesized by *Bifidobacterium longum* decreased blood glucose, body weight, and mitigated early diabetes progression [100]. Also, a spherical phytogenic Se-NPs sized 100 nm bioformed from *Caralluma tuberculata* showed a hypoglycemic effect against α-amylase (78.2%) and α-glucosidase (71.6%) [101].

The antidiabetic properties of Se-NPs were attributed to their antioxidant effects. In particular, Se-NPs have been demonstrated to increase several enzymes, including glutathione peroxidase (GPx), catalase (CAT), and superoxide dismutase (SOD) activities. Consequently, such an increase in antioxidant enzyme activity leads to a decline in low-density lipoprotein cholesterol (LDL-C), triglycerides, and total cholesterol levels while simultaneously elevating high-density lipoprotein (HDL) cholesterol levels [102]. These findings suggest that Se-NPs can be considered potential antidiabetic adjuncts due to their ability to modulate oxidative stress and lipid metabolism, which play a critical role in the development and progression of diabetes mellitus.

#### Antimicrobial activity

##### Antifungal activity

**Fig. 6:**
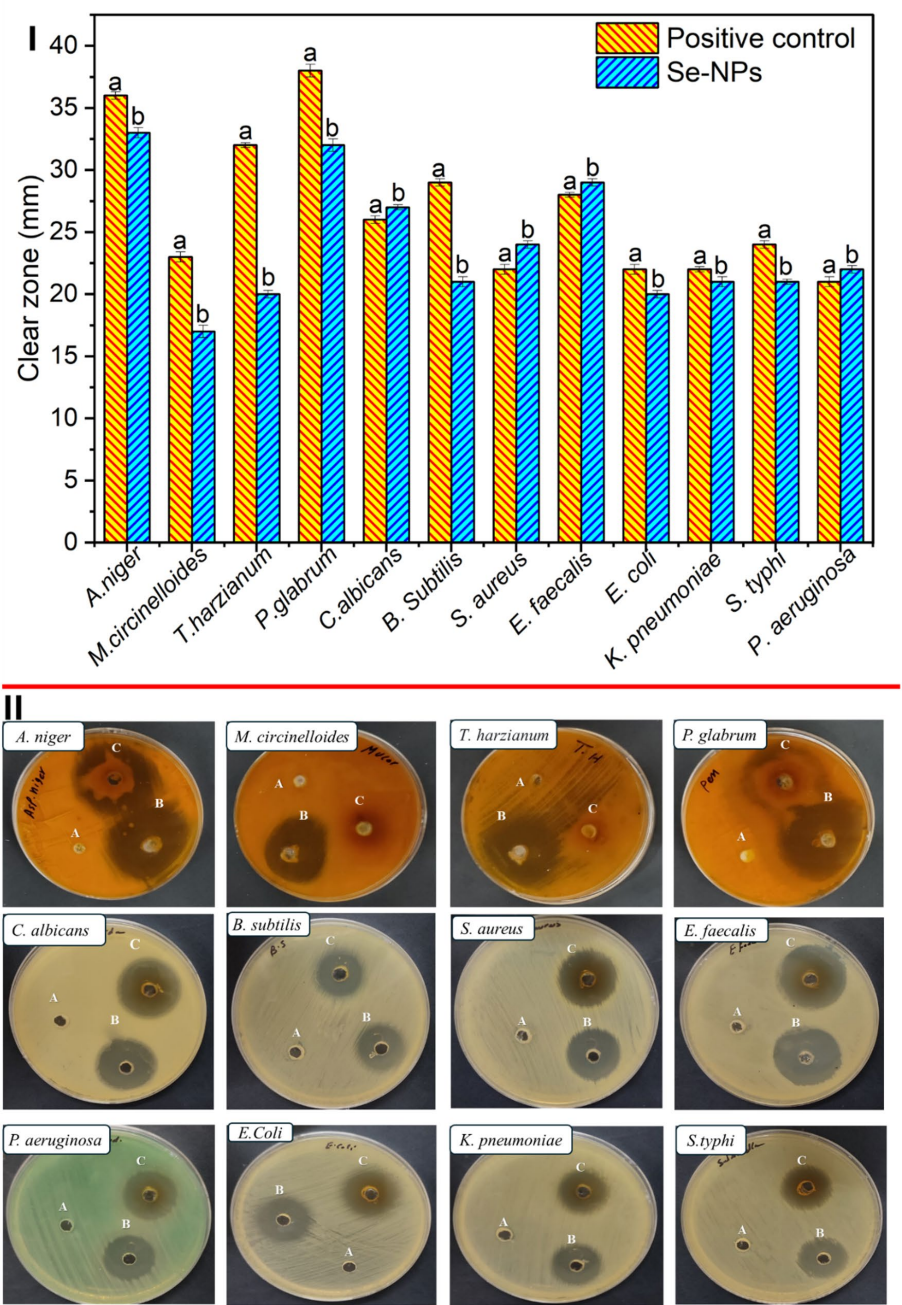
Antimicrobial activity of Se-NPs (10 mg mL^–1^) against pathogenic multicellular and unicellular fungi, and Gram-positive and Gram-negative bacteria. (**I**) Statistical analysis of 10 mg mL^–1^ against tested strains and represented the data by inhibition zone (mm), (**II**) Photo of plates showing the inhibition zones for DMSO as negative control (**A**), fluconazole as a positive control of fungi and gentamicin as a positive control for bacteria (**B**), and Se-NPs (**C**). Different letters (**a** and **b**) on the bars indicate the data (inhibition zones) are significantly different (*n* = 3, *P* ≤ 0.05)

Se-NPs demonstrated comparable or slightly lower antifungal activity against multicellular tested fungi compared to positive control. The inhibition zone of Se-NPs (33 ± 0.4 mm) against *Aspergillus niger* was slightly smaller than fluconazole (36 ± 0.3 mm). Similarly, against *Penicillium glabrum*, Se-NPs (32 ± 0.5 mm) showed a marginally lower inhibition zone than fluconazole (38 ± 0.5 mm). However, while fluconazole produced an inhibition zone of 23 ± 0.4 mm against *Mucor circinelloides*, Se-NPs exhibit inhibition zone of 17 ± 0.5 mm. For *Candida albicans*, Se-NPs (27 ± 0.2 mm) had a significant inhibition zone compared to fluconazole (26 ± 0.3 mm). Overall, Se-NPs exhibited notable antifungal activity, as evidenced by the largest inhibition zone observed against *Aspergillus niger* (33 ± 0.4 mm), followed closely by *Penicillium glabrum* (32 ± 0.5 mm), suggesting potent activity against these two filamentous fungi. Moreover, *Candida albicans* also showed a considerable inhibition zone (27 ± 0.2 mm), indicating good antifungal activity against this yeast species. In contrast, *Trichoderma harzianum* was only moderately inhibited, with an inhibition zone of 20 ± 0.3 mm (Fig. 6I and II).

Regarding the MICs, Se-NPs were most effective against *Aspergillus niger* (MIC = 25 µg mL^–1^) and *Candida albicans* (MIC = 12.5 µg mL^–1^), implying higher susceptibility of these species. *Trichoderma harzianum* had the highest MIC (100 µg mL^–1^), suggesting lower susceptibility. Regarding MFCs, the lowest value was observed for *Candida albicans* (50 µg mL^–1^), indicating that Se-NPs can effectively kill this yeast at a relatively low concentration. However, higher concentrations (200 µg mL^–1^) were required to achieve fungicidal activity against *Aspergillus niger* and *Penicillium glabrum* (Table 4).

**Table 4:** MIC, MBC, MFC, and their ratios (MBC/MIC and MFC/MIC) of Se-NPs against various bacterial and fungal strains

Tested bacterial strains	MIC (µg/mL)	MBC (µg/mL)	MBC/MIC Ratio	Tested filamentous Fungi and yeasts	MIC (µg/mL)	MFC (µg/mL)	MFC/MIC ratio
*Bacillus Subtilis*	100	300	3	*Aspergillus niger*	6.25	200	32
*Staphylococcus aureus*	50	200	4	*Mucor circinelloides*	100	300	3
*Enterococcus faecalis*	25	50	2	*Trichoderma harzianum*	100	200	2
*Escherichia coli*	100	200	2	*Penicillium glabrum*	50	200	16
*K. pneumoniae*	100	200	2	*Candida albicans*	12.5	50	4
*Salmonella typhi*	100	200	2				
*Pseudomonas aeruginosa*	50	50	1				

The spherical Se-NPs (20–80 nm) synthesized by *Streptomyces vinaceusdrappus* AMG31 exhibited differential antifungal activity through multiple mechanistic pathways. The nanoparticles’ crystalline structure (evidenced by XRD peaks at 23.4°, 30.13°, 41.38°) enables penetration through fungal cell walls, while their negative surface charge (−37.9 mV) facilitates strong electrostatic interactions with positively charged components of fungal membranes, disrupting membrane integrity [103]. Against *Aspergillus niger* and *Penicillium glabrum*, Se-NPs demonstrate remarkable efficacy (MIC 6.25 µg mL^–1^ and 50 µg mL^–1^, respectively), likely due to selenium-induced oxidative stress that triggers lipid peroxidation in fungal membranes [104].

The high MFC/MIC ratio for *A. niger* (32) suggests Se-NPs initially inhibit growth at low concentrations by disrupting critical enzymes containing sulfhydryl groups, while complete fungicidal effects require higher concentrations to overwhelm cellular defense mechanisms [105]. The functionalization of Se-NPs with protein and polysaccharide capping agents (confirmed by FT-IR peaks at 3380 cm⁻¹, 1685 cm⁻¹, and 1000–1200 cm⁻¹) enhances their interaction with chitin and β-glucan components of fungal cell walls [106]. Notably, Se-NPs’s low effectiveness against *Mucor circinelloides* correlates with this organism’s unique cell wall composition, suggesting Se-NPs’s antifungal mechanisms are species-dependent and influenced by target organism physiological characteristics rather than following a universal pathway.

Se-NPs generate ROS in fungal cells, causing oxidative stress that damages DNA, proteins, and lipids. Studies show these nanoparticles elevate ROS levels, triggering lipid peroxidation and membrane breakdown [107]. Simultaneously, Se-NPs decrease antioxidant enzyme activity, intensifying oxidative damage and creating a dual-action mechanism that enhances their antifungal effectiveness [108]. Physical aggregation is another key antifungal mechanism of Se-NPs, as it immobilizes fungi by adhering to their zoospores, preventing movement and plant invasion [44]. This surface-level interaction disrupts spore function and infectivity, complementing the biochemical antifungal activities and creating a comprehensive defence system against fungal pathogens. Recently, Gharieb and coauthors reported that the Se-NPs penetrate into fungal cells, accumulated inside, and destroyed essential cellular structures as shown by TEM analysis [105].

Also, Se-NPs target fungal genetic material directly, twisting DNA into unusable forms and disrupting DNA integrity, suppressing cellular division, which is critical for fungal survival and reduces pathogen viability [109]. Furthermore, Se-NPs disrupt fungi’s critical RAS/cAMP/PKA signalling cascade, blocking cellular communication networks required for growth and biofilm development [110]. This interference impairs fungal reproduction and prevents the establishment of treatment-resistant colonies.

##### Antibacterial activity

Se-NPs demonstrated considerable antibacterial activity against all three Gram-positive bacteria, with different degrees of potency. While gentamicin showed more potent inhibition against *B. subtilis*, Se-NPs outperformed gentamicin against *S. aureus* and *E. faecalis* regarding inhibition zones. The lowest MIC and MBC values and the lowest MBC/MIC ratio were observed for *E. faecalis*, suggesting that Se-NPs may be particularly effective against this pathogen. The inhibition zone produced by Se-NPs against *B. subtilis* was 21 ± 0.4 mm, which is lower than the control antibiotic gentamicin (29 ± 0.3 mm), suggesting that gentamicin has stronger antibacterial activity against this bacterium. The MIC value for Se-NPs against *B. subtilis* was 100 µg mL^–1^, while the MBC value was 300 µg mL^–1^, resulting in an MBC/MIC ratio of 3 (Table 4). Against *S. aureus*, Se-NPs showed a slightly larger inhibition zone (24 ± 0.3 mm) compared to gentamicin (22 ± 0.4 mm), indicating that Se-NPs may have better diffusion or antibacterial activity against this pathogen (Fig. 6I and II).

The MIC value for Se-NPs against *S. aureus* was 50 µg mL^–1^, which is lower than that of *B. subtilis*, implying that *S. aureus* is more susceptible to Se-NPs. However, the MBC value was higher at 200 µg mL^–1^, resulting in an MBC/MIC ratio of 4 (Table 4). Additionally, Se-NPs exhibited the largest inhibition zone (29 ± 0.3 mm) against *E. faecalis* among the three G + ve bacteria, which was slightly higher than gentamicin (28 ± 0.2 mm) (Fig. 6I and II). This indicates that Se-NPs have potent antibacterial activity against *E. faecalis*. Furthermore, Se-NPs had the lowest MIC value of 25 µg mL^–1^and the lowest MBC value of 50 µg mL^–1^ against *E. faecalis*, resulting in an MBC/MIC ratio of 2 (Table 4). This relatively low ratio suggests that the concentration required for bactericidal activity is only twice that of inhibition, making Se-NPs an effective antibacterial agent against *E. faecalis* (Table 4).

Concerning the G-ve tested bacteria, in terms of inhibition zones, Se-NPs exhibited the largest zone against *Pseudomonas aeruginosa* (22 ± 0.3 mm), outperforming the control antibiotic gentamicin (21 ± 0.4 mm). *Escherichia coli* had the smallest inhibition zone (20 ± 0.3 mm) among the G-ve bacteria, slightly smaller than gentamicin (22 ± 0.4 mm). *Klebsiella pneumoniae* and *Salmonella typhi* showed similar inhibition zones (21 ± 0.4 mm and 21 ± 0.2 mm, respectively), which were marginally smaller than gentamicin (Fig. 6I and II).

Regarding MIC and MBC values, *P. aeruginosa* exhibited the lowest MIC (50 µg mL^–1^) and MBC (50 µg mL^–1^) among the G-ve bacteria, resulting in the most favorable MBC/MIC ratio of (1) This ratio indicates that the concentration required for bactericidal activity is the same as the concentration needed for inhibition, making Se-NPs a highly effective antimicrobial agent against *P. aeruginosa*. In contrast, *E. coli*, *K. pneumoniae*, and *S. typhi* all had higher MIC and MBC values of 100 µg mL^–1^and 200 µg mL^–1^, respectively, with an MBC/MIC ratio of (2) Among all tested bacteria, Se-NPs exhibited the most potent activity against *E. faecalis*, with the largest inhibition zone, lowest MIC and MBC values, and a MBC/MIC ratio of 2. Also, *P. aeruginosa* was the most susceptible to Se-NPs, with the largest inhibition zone, lowest MIC and MBC values, and an exceptional MBC/MIC ratio of 1, indicating high bactericidal efficacy (Table 4).

In accordance with our findings, spherical mycofabricated Se-NPs sized 110 nm from *Yarrowia lipolytica* showed antibacterial activity at a concentration of 160 µg mL^–1^ against *K. pneumonia* and *E. coli* [111]. Bacterial synthesized Se-NPs from *Shewanella* sp. were reported to be spherical and sized 20 nm, demonstrated antimicrobial activity against *P. aeruginosa* with MIC at 8 µg/200 mL and MBC at 16 µg/100 mL by micro broth dilution method [112]. A spherical mycofabricated Se-NPs from *Penicillium chrysogenum* sized 78 nm was reported to inhibit the bacterial growth of *S*. *aureus* with MIC Value = 0.31 mg mL^–1^ [79]. Similarly, *Talaromyces haitouensis* was employed to fabricate a Se-NPs that was spherical sized 200 nm that had a bacteriostatic impact on *S. aureus* and *E. coli* with reported MIC = 125 µg mL^–1^through micro broth dilution technique [113]. Also, a bacterial synthesized Se-NPs from *Lactobacillus plantarum* with an average size of 270 nm was reported to have a bactericidal action against *S. mutans* with a MIC of 3.1 mg mL^–1^ [114].

The current biogenic SeNPs from *Streptomyces vinaceusdrappus* AMG31 demonstrated potent antibacterial activity due to their physicochemical attributes. These spherical nanoparticles (20–80 nm) featuring crystalline structure, negative surface charge (−37.9 mV), and stable dispersion (PDI 0.384) effectively disrupted bacterial cells, with particular efficacy against *Enterococcus faecalis* (Gram-positive) and *Pseudomonas aeruginosa* (Gram-negative), achieving MBC/MIC ratios of 2 and 1, respectively. The SeNPs compromise cytoplasmic membrane integrity through direct physical damage and by generating ROS that oxidize membrane lipids [115]. Additionally, the nanoparticles facilitate gradual release of Se ions that accumulate within bacterial cells to toxic levels, disrupting vital metabolic processes by binding to thiol groups in proteins and enzymes [116]. The functional biomolecules identified by FT-IR (amines, phenols, polysaccharides) capping the SeNPs contribute synergistically to the antibacterial activity by modifying surface properties and enhancing stability, thereby prolonging interaction time with bacterial cells [117].

Furthermore, the antimicrobial impact of Se-NPs originates from their capacity to cause metabolic impairment through decreasing energetic levels. This results in an elevation of ROS, which over stresses the antioxidant defences of the bacterial cell [118]. Such augmented oxidative stress disrupts bacterial defense mechanisms. Furthermore, selenium nanoparticles interfere with protein synthesis, impede protein translation, and induce mutations in the microbes’ genetic material, thus disturbing critical functions of a cell. Se-NPs also induce depolarization and lysis of the bacterial cell membrane, which leads to the release of crucial cellular contents and, eventually, the death of the microbial cell [115].

Also, biogenic Se-NPs attack bacteria by binding directly to -SH groups in metabolic enzymes to halt energy production while simultaneously degrading membrane structure through phospholipid interactions [119]. This two-pronged approach causes cellular contents to leak and ultimately destroys bacterial cells, including those with antibiotic-resistance mechanisms. Moreover, Se-NPs block bacterial attachment and quorum sensing signals, halting biofilm development. Their nanoscale dimensions allow penetration through established biofilms, where they break down the EPS matrix and expose protected bacteria to elimination [120].

#### Antibiofilm potential of Se-NPs

Se-NPs showed potent antibiofilm activity against the tested G + ve bacteria, with varying degrees of inhibition at different concentrations of MBCs. Among the three bacteria, *S. aureus* showed the highest antibiofilm activity, with 90.9% inhibition at 75% of the MBC, followed by *E. faecalis* with 90.2% inhibition at the same concentration. *B. subtilis* exhibited the lowest antibiofilm activity at 75% of the MBC, with 79.7% inhibition. Interestingly, for *S. aureus*, even at 50% of the MBC, Se-NPs achieved a remarkable 85.8% antibiofilm activity, suggesting its high efficacy against this pathogen. In contrast, for *B. subtilis* and *E. faecalis*, the antibiofilm activity at 50% of the MBC was lower, at 71.9% and 80.2%, respectively. At 25% of the MBC value, *S. aureus* again demonstrated the highest antibiofilm inhibition of 76.3%, followed by *E. faecalis* by percentages of 68.4% and *B. subtilis* at 63.4%.

**Fig. 7:**
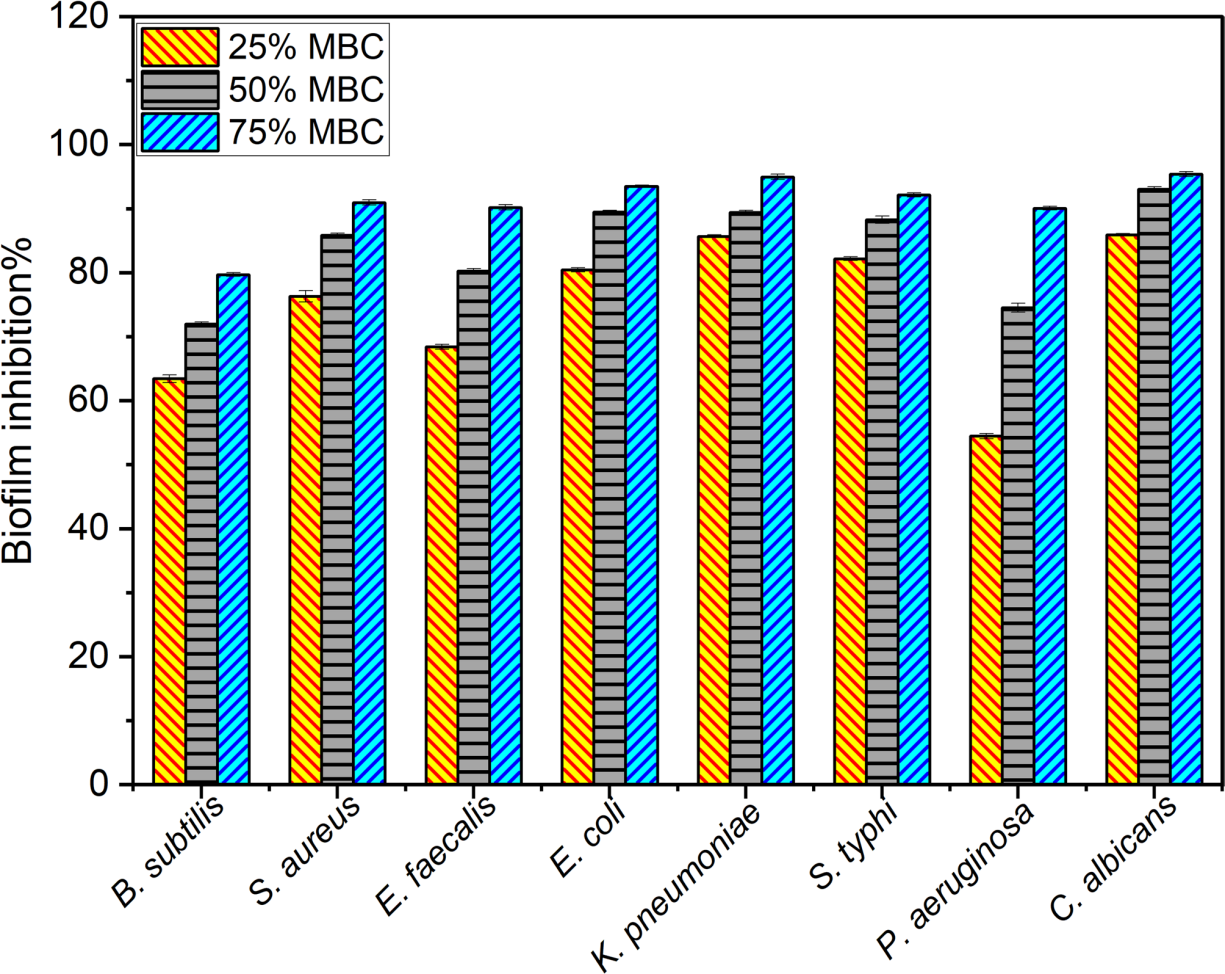
Antibiofilm activity of Se-NPs against different ATCC pathogenic bacterial strains and *C. albicans* at different 25, 50, and 75% of MBC/MFC value

The results of the G-ve antibiofilm inhibition also confirmed the potent antibiofilm activity of Se-NPs, where *K. pneumoniae*, *E. coli*, and *S. typhi* were highly susceptible, showing over 90% inhibition at 75% of the MBC. While *P. aeruginosa* was the least susceptible, *Klebsiella pneumoniae* exhibited the highest antibiofilm activity, with 94.95% inhibition observed at 75% MBC. This was followed closely by *E. coli*, which showed 93.5% inhibition at the same concentration. *Salmonella typhi* also demonstrated excellent antibiofilm activity, with 92.1% inhibition at 75% of the MBC. In contrast, *P. aeruginosa* showed the lowest antibiofilm activity at 75% of the MBC, with 90.1% inhibition. However, it is noteworthy that *P. aeruginosa* exhibited the highest resistance to the antibiofilm effects of Se-NPs, as even at 25% of the MBC, the inhibition was only 54.4%, which is Significantly lower compared to the other G-ve bacteria tested. At 50% of the MBC, *K. pneumoniae* and *E. coli* showed similar levels of antibiofilm activity, with 89.4% and 89.4% inhibition, respectively. *Salmonella typhi* also exhibited a high antibiofilm activity of 88.25% at this concentration (Fig. 7) (Table S1 & S2, See supplementary data).

Se-NPs was further investigated as an anti-candidal biofilm agent, that clinically relevant yeast pathogen known for its ability to form biofilms and cause persistent infections [121]. At 75% of the MBC, Se-NPs achieved an impressive 95.4% inhibition of *C. albicans* biofilm formation. This high level of antibiofilm activity suggests that Se-NPs is highly effective against this pathogenic yeast at relatively lower concentrations. Even at 50% of the MBC, Se-NPs demonstrated a remarkable 93.03% antibiofilm activity against such opportunistic pathogenic yeast. At the lowest concentration tested, 25% of the MBC, Se-NPs still exhibited a considerable 85.9% inhibition of *C. albicans* biofilm formation. While lower than the higher concentrations, this activity level is notable and indicates that Se-NPs retains significant antibiofilm properties and could be a promising candidate for developing antibiofilm strategies targeting *C. albicans* biofilms even at lower concentrations (Fig. 7).

The comparatively low antifungal activity of Se-NPs when compared to fluconazole may be related to the thick cell wall structure of fungi mainly made up of complex polysaccharides and proteins which hinder the penetration and efficacy of the nanoparticles. On the other hand, Se-NPs showed good level of antibacterial and biofilm inhibition against both G + ve and G-ve bacteria with different efficacy. The aforementioned broad spectrum of antibacterial activity could be attributed to several key factors. Firstly, the small size of the Se-NPs, around 20–80 nm, and their negative surface charge of −25.6 mV promote entrance and bonding with the bacterial cell membrane which eventually leads to the membrane integrity disruption and efflux of microbial cellular contents. Secondly, the overproduction of ROS brought by Se-NPs triggered oxidative stress that surpassed the bacteria’s antioxidant responses and led to cell injury.

Additionally, Se-NPs inhibited protein synthesis and DNA replication, essential functions of the cell, eventually resulting in the bacterial cell’s death [122].

Notably, G + ve bacteria were significantly more susceptible to the antimicrobial effects of Se-NPs than G-ve species. This can be explained by the differences in the composition of the cell walls of the two groups. Although the peptidoglycan layer in G + ve bacteria is thicker, it’s more porous than that in G-bacteria [123], consequently allowing better penetration and rapid interaction of Se-NPs with the cytoplasmic membrane. On the contrary, the Se-NPs cannot penetrate and act freely in G-ve bacteria due to the presence of an additional outer membrane layer composed of lipopolysaccharides (LPS) which is a major permeability barrier. Moreover, the electrostatic repulsion due to the negatively charged Se-NPs and the negatively charged LPS present on the outer membrane reduces the inhibitory activity of the Se-NPs against the G-ve bacterial strains [106].

#### Anti-inflammatory assay

**Fig. 8:**
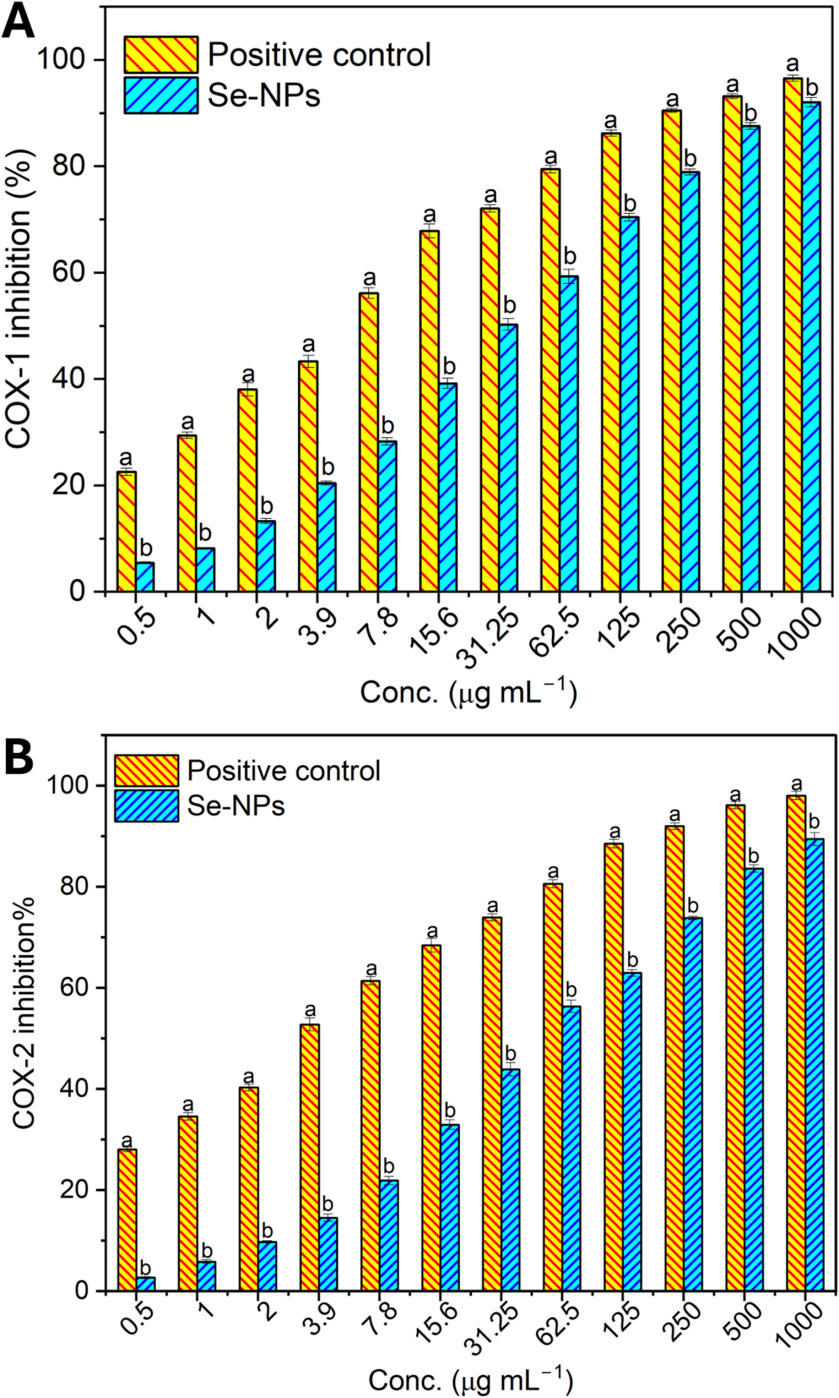
Anti-inflammatory activity of Se-NPs vs. positive control (Celecoxib) at various concentrations through inhibition of COX-1 and COX-2 enzyme activity. Data represented as Mean ± SD (*n* = 3, *P* ≤ 0.05). Different letters (**a** and **b**) on the bars indicate the data (inhibition percentages) are significantly different

Se-NPs anti-inflammatory testing showed that both COX-1 and COX-2 enzymes were significantly inhibited, with the Se-NPs appearing to be slightly more selective towards COX-1 inhibition. Responses at various concentrations were evident, as the inhibition of COX-1 detected was between 5.4% and 92.03% at concentrations of 0.5–1000 µg mL^–1^ with an IC_50_ of 30.9 ± 1.02 µg mL^–1^ (Fig. 8A). Inhibition of COX-2 at the same concentration range was from 2.7 to 89.4%, the IC_50_ was 46.6 ± 1.3 µg mL^–1^ when compared to Celecoxib IC_50_ of 5.9 ± 0.4 µg mL^–1^ for COX-1 and 3.6 ± 0.2 µg mL^–1^ for COX-2 (Fig. 8B).

Se-NPs achieved potent inhibition at higher concentrations, particularly at 1000 µg mL^–1^, where 92.03% COX-1 and 89.4% COX-2 inhibition were recorded. At 500 µg mL^–1^, the activity observed was consistently strong at 87.6% and 83.6%, respectively, for COX-1 and COX-2. The rates were equally considerable at 250 µg mL^–1^, with 78.9% COX-1 and 73.8% COX-2 inhibition (Fig. 8A and B).

COX-enzymes are key enzymes that catalyze prostaglandin production, intensifying inflammation. Se-NPs demonstrate potent anti-inflammatory action by blocking COX-2 expression via several pathways. These nanoparticles diminish inflammatory mediators through the simultaneous reduction of COX-2 and inducible nitric oxide synthase (iNOS) levels [75]. SeNPs also block key pro-inflammatory cytokines, such as TNF-α, IL-1β, IL-6, and MCP-1, that maintain inflammation and drive COX-2 upregulation [124].

Se-NPs combat inflammation through multiple mechanisms, they suppress ROS production that triggers inflammatory cascades and COX-2 expression; boost antioxidant enzymes glutathione peroxidase (GPx) and superoxide dismutase (SOD) that neutralize ROS [17], and block key signalling pathways of NF-κB and p38/MAPK that control COX-2 expression [125]. These diverse actions have proven effective against osteoarthritis [126], rheumatoid arthritis [127], and psoriasis [128], highlighting Se-NPs as promising therapeutics that target upstream inflammatory processes to reduce COX-2 activity.

## Conclusion

The present study effectively biosynthesized Se-NPs using the biomass filtrate of the actinobacterium *Streptomyces vinaceusdrappus* AMG31. The biosynthesized Se-NPs exhibited noteworthy antioxidant, wound healing, anticancer, biocompatibility, antidiabetic, hemocompatibility, antimicrobial, antibiofilm, and anti-inflammatory properties. Their selective cytotoxicity towards cancer cells and ability to inhibit enzymes involved in carbohydrate metabolism suggests their potential as anticancer and antidiabetic agents. Furthermore, the potent antimicrobial and antibiofilm activities against a wide range of pathogenic bacteria and fungi highlight the therapeutic potential of these green-synthesized Se-NPs as antimicrobial and antibiofilm agents. Also, its activity in inhibiting COX-1 and COX-2 enzymes at different concentrations with varied percentages indicates their anti-inflammatory effectiveness. Collectively, the multifaceted bioactivities exhibited by these biosynthesized Se-NPs, highlight their potential for further development as therapeutic and biomedical nanomaterials. Future investigation should expand this research through in vivo studies to validate Se-NPs’ effects in biological systems and establish appropriate dosing regimens. Assessing long-term stability under various storage conditions would determine shelf-life parameters essential for practical applications. Detailed molecular analysis using genomics and proteomics could illuminate the specific signaling pathways and protein targets underlying the observed anticancer, antimicrobial, and antidiabetic activities.

## Supplementary Information

Supplementary Material 1.

## Data Availability

The datasets generated and/or analyzed during the current study are available upon request from the corresponding authors.
