# Supplementary material for: Marine actinobacterium Streptomyces vinaceusdrappus mediated nano-selenium: biosynthesis and biomedical activities
Source: BMC Complement Med Ther. 2025 Sep 17;25:329. doi: 10.1186/s12906-025-05073-9 (PMC12442295; doi:10.1186/s12906-025-05073-9)
Supplement: Supplementary file 1 — Supplementary Material 1. [file 12906_2025_5073_MOESM1_ESM.docx]

**Supplementary Data**

**Marine Actinobacterium *Streptomyces vinaceusdrappus* Mediated Nano-Selenium: Biosynthesis and Biomedical Activities**

**Ahmed Ghareeb** ^1^**, Amr Fouda** ^2*^**, Rania M. Kishk** ^3^ **and Waleed M. El Kazzaz** ^1^

^1^ Botany and Microbiology Department, Faculty of Science, Suez Canal University, Ismailia 41522, Egypt.

^2^ Botany and Microbiology Department, Faculty of Science, Al-Azhar University, Nasr City, Cairo 11884, Egypt.

^3^ Microbiology and Immunology Department, Faculty of Medicine, Suez Canal University, Ismailia 41522, Egypt.

^*^ Corresponding author: Amr Fouda, [amr_fh83@azhar.edu.eg](mailto:amr_fh83@azhar.edu.eg) (ORCID Number: [https://orcid.org/0000-0003-3840-7837](https://www.scopus.com/redirect.uri?url=https://orcid.org/0000-0003-3840-7837&authorId=57194940078&origin=AuthorProfile&orcId=0000-0003-3840-7837&category=orcidLink))

- **FT-IR for actinomycetes biomass filtrate.**


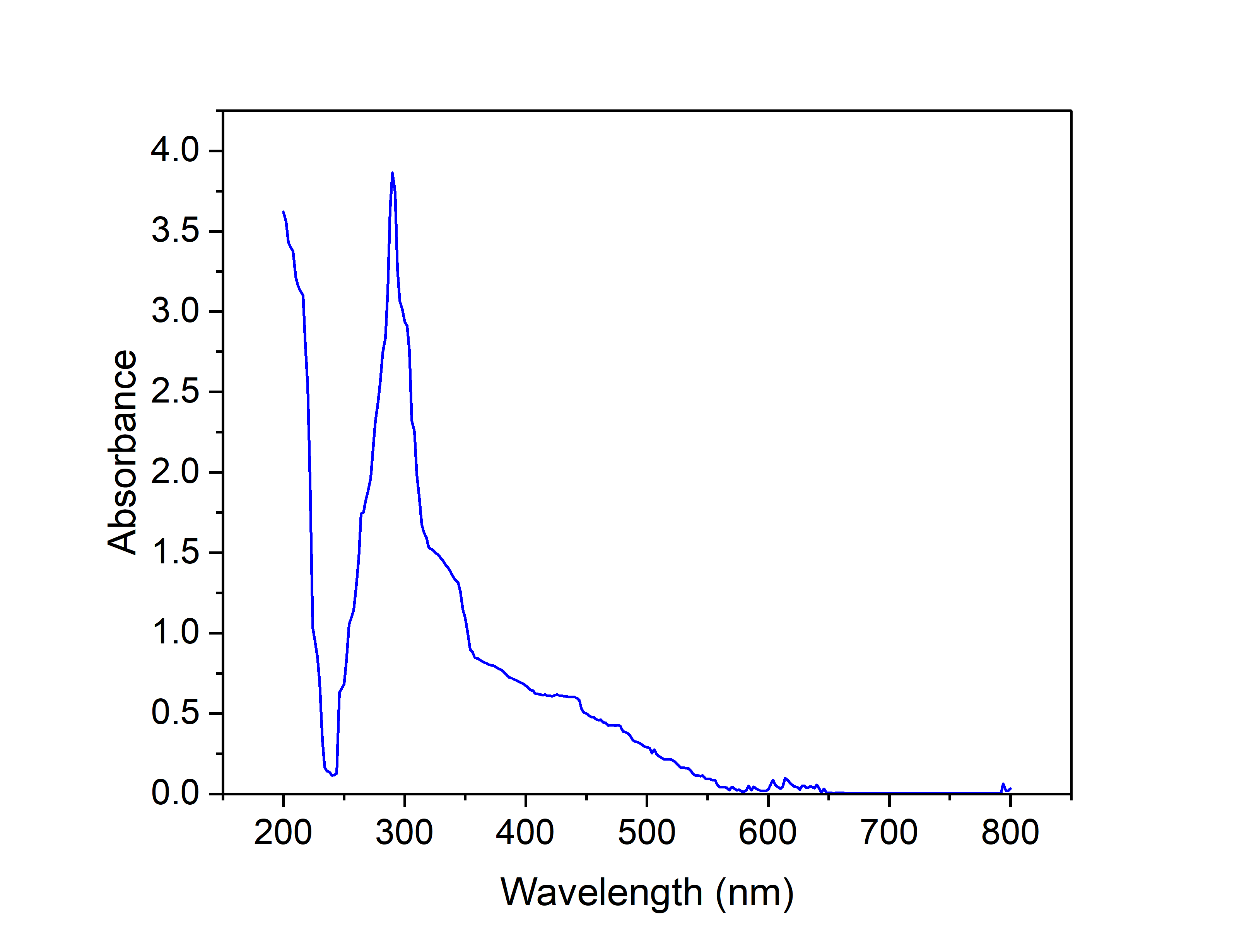


Fig. S1. UV chart for actinomycetes biomass filtrate.

- **
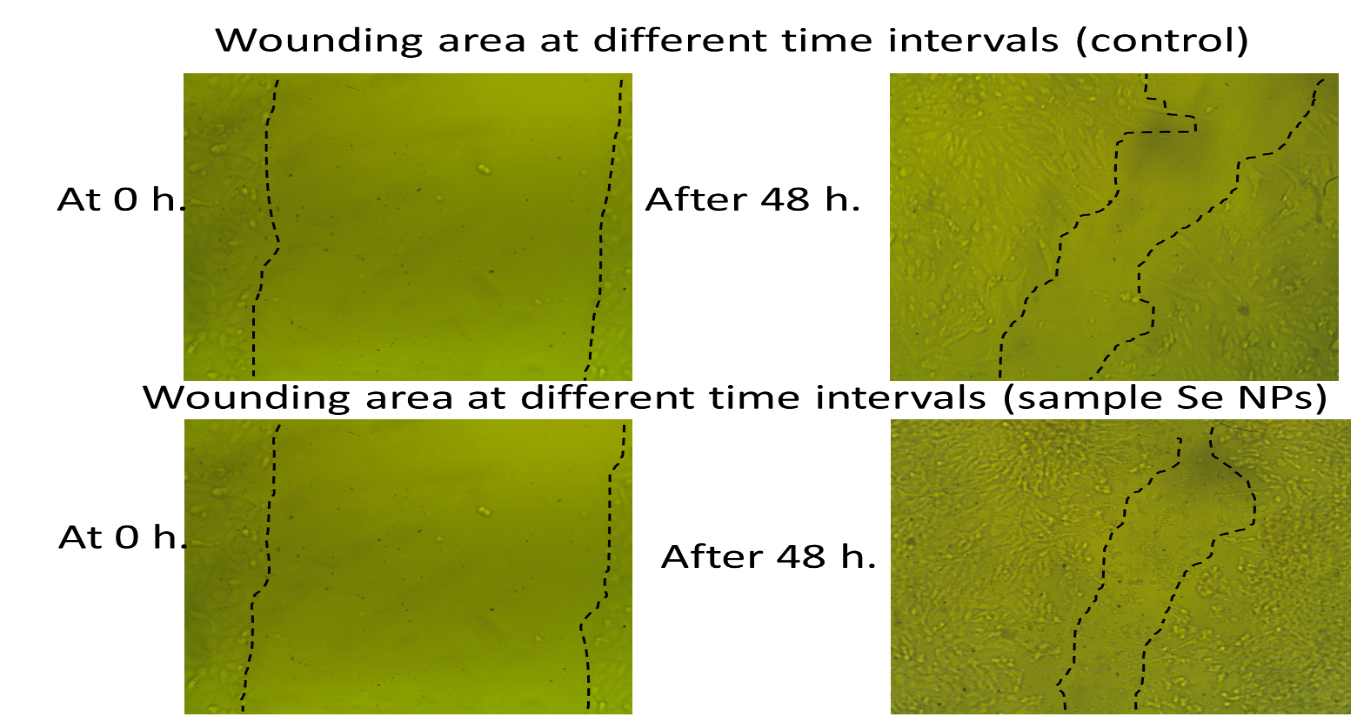
Wound Healing**

Fig.S2: Comparative effect of Se-NPs (209.87 µg mL^–1^) and control on wound closure area of HFB4 cell line at different time intervals. (A) Untreated cells at 0 h, (B) Treated cells with control after 48 h, (C) Untreated cells at 0 h, and (D) Treated cells with Se-NPs after 48 h

- **Hemocompatibility examination of SeNP**


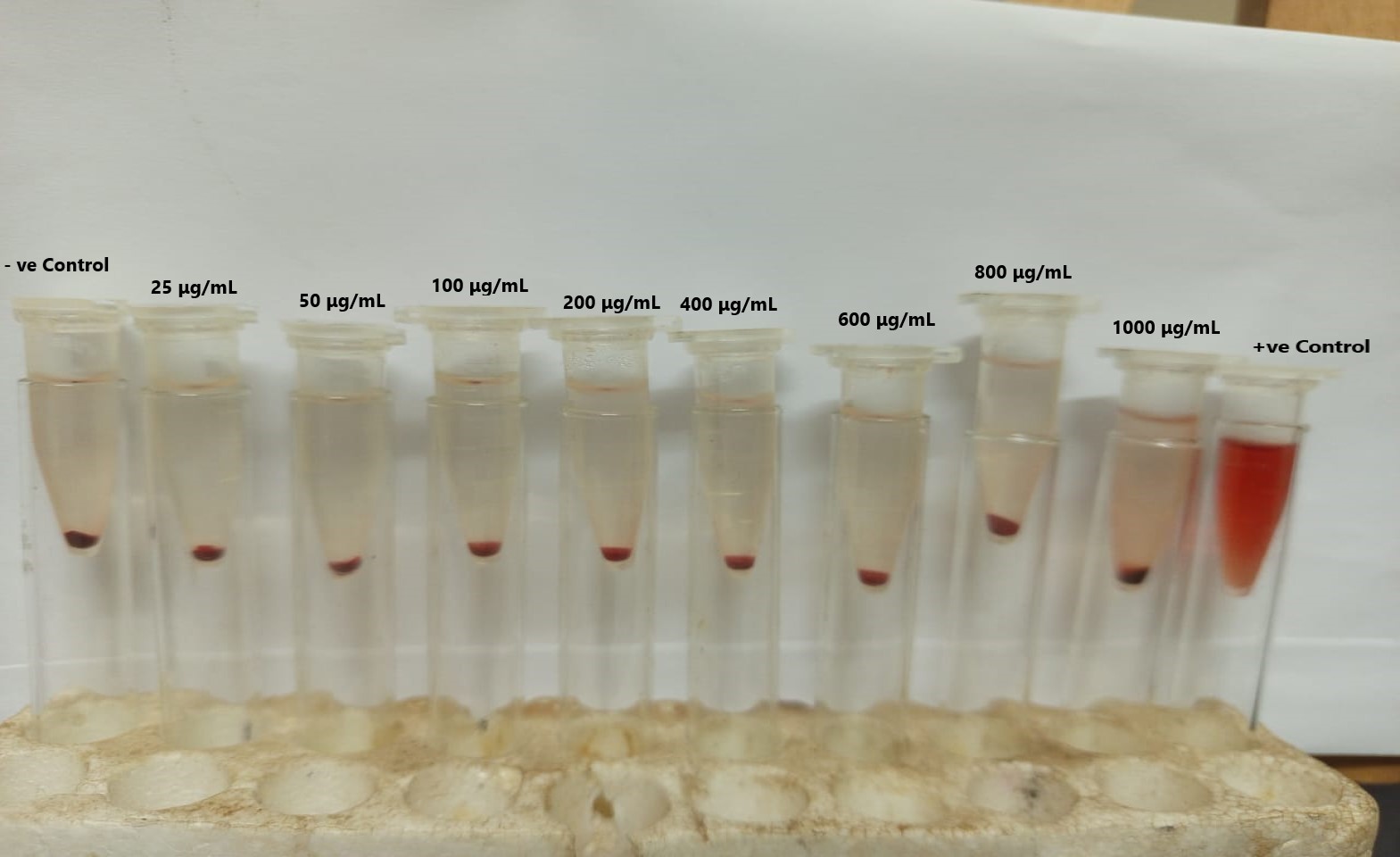


Fig。 S3. The concentration gradient of SeNPs and its effects on RBCs hemolysis

- **Cytotoxicity and Anticancer activity of** **SeNP**


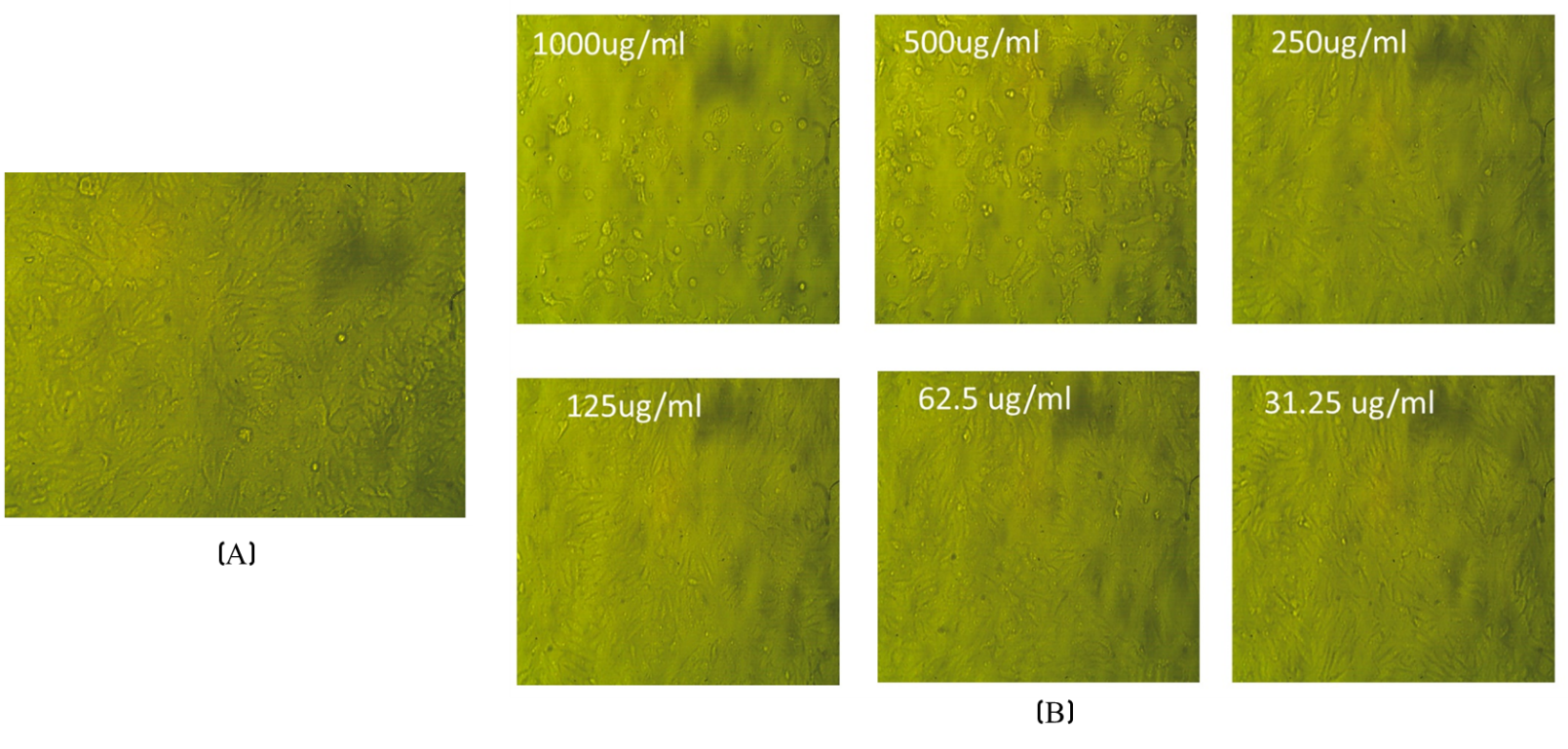


Fig。 S4: Morphological changes and size variation of WI-38 cells before and after SeNP treatment (A) Untreated WI38 cells showing normal morphology and size (B) WI-38 cells treated with 31.25-1000 μg/mL SeNP.


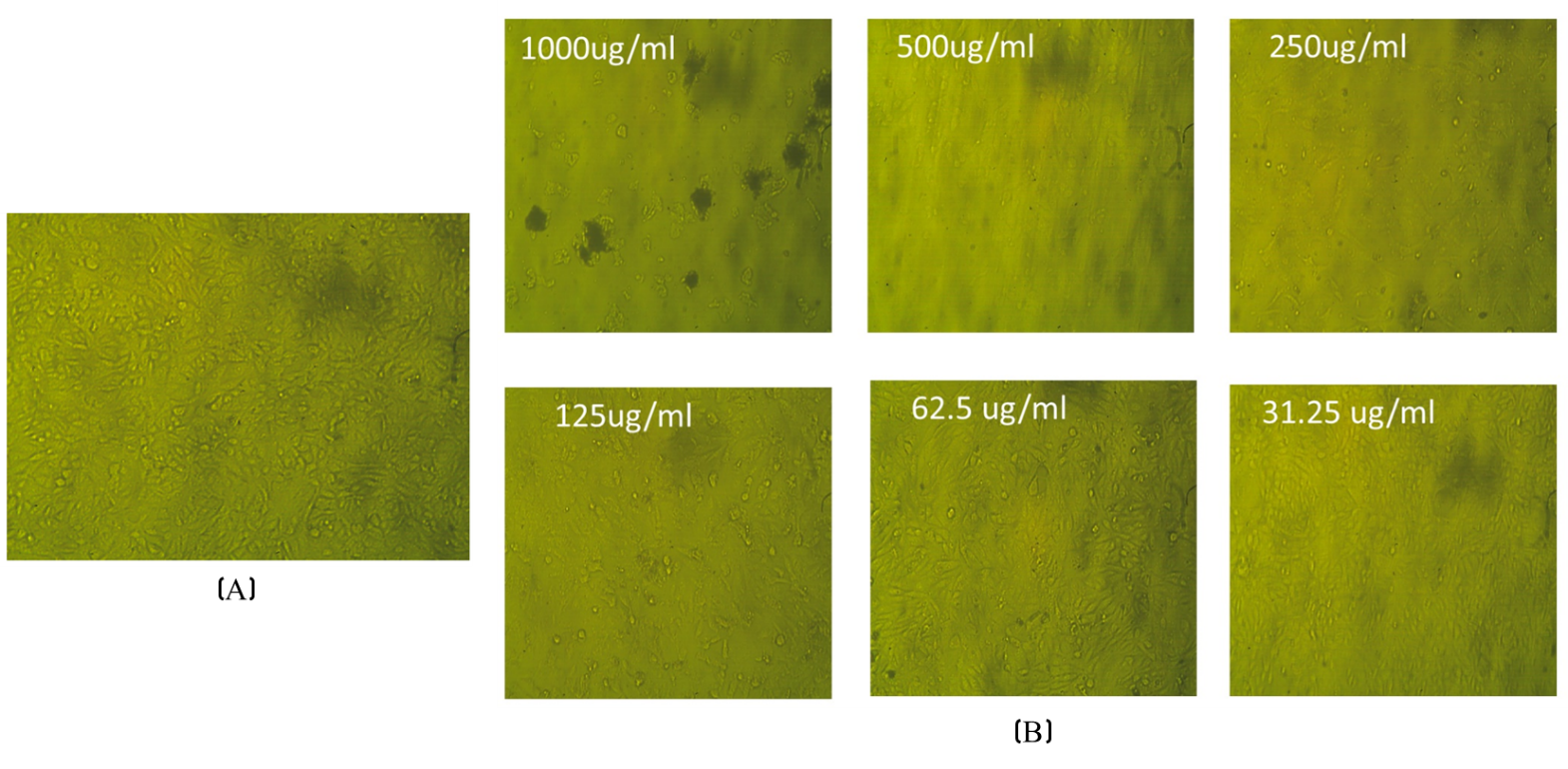


Fig。 S5: Morphological changes and size variation of Caco-2 cancer cell line before and after Se-NP treatment (A) Untreated Caco-2 cancer cell line showing normal morphology and size (B) Caco-2 cancer cell line treated with 31.25-1000 μg/mL Se-NP showing changes in morphology and size.


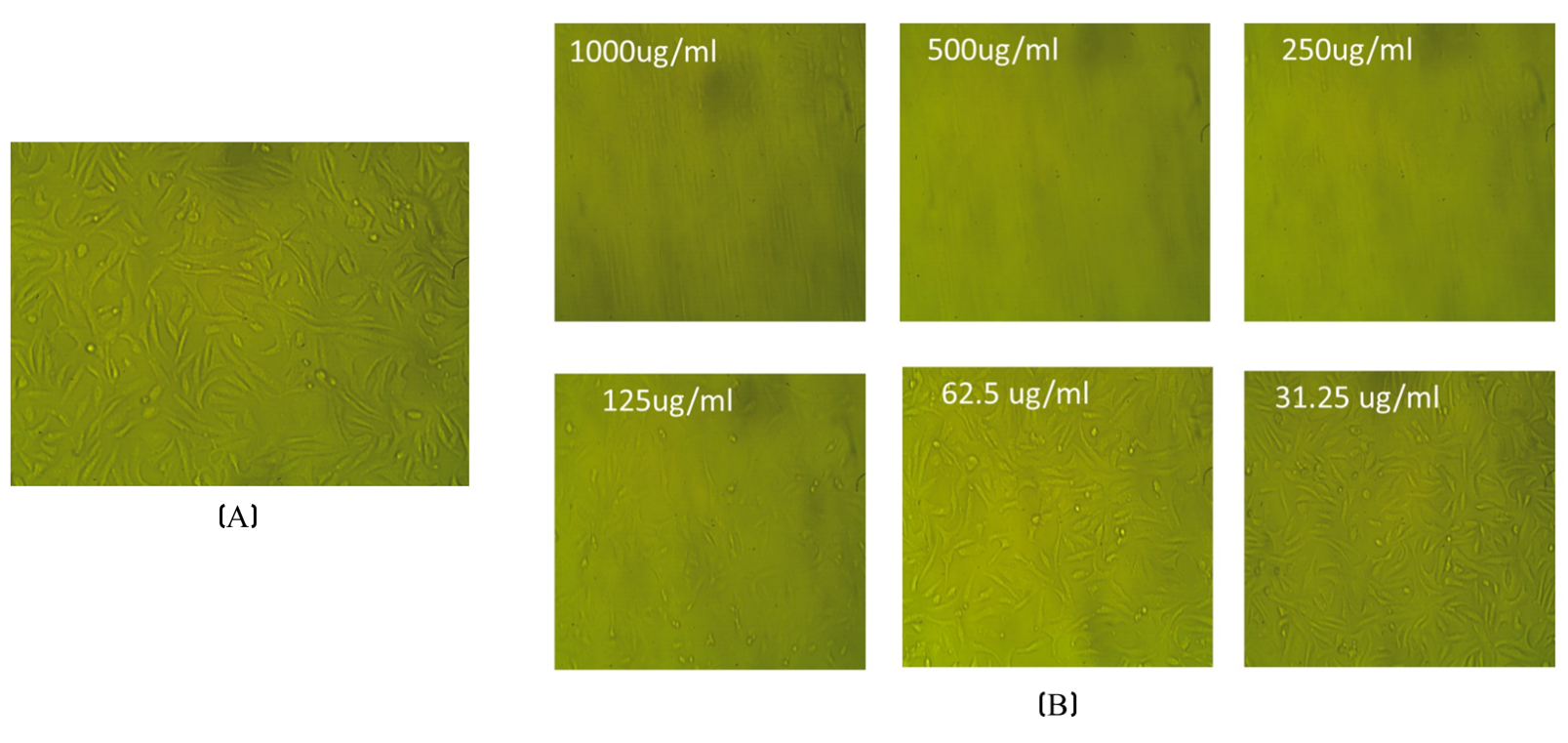


Fig。 S6: Morphological changes and size variation of PANC-1 cancer cell line before and after SeNP treatment (A) Untreated PANC-1 cancer cell line showing normal morphology and size (B) PANC-1 cancer cell line treated with 31.25-1000 μg/mL Se-NP showing changes in morphology and size.

**-Antibiofilm Activity of SeNP**

| **SeNP - MBC% of**  ***E. faecalis* Anti-biofilm %** |
| --- |
| Blank (Media only) - |
| Media+ Organism (Cont.) - |
| 25% of MBC 68.39 |
| 50% of MBC 80.22 |
| 75% of MBC 90.18 |
| **SeNP - MBC% of**  ***B. Subtilis* Anti-biofilm %** |
| Blank (Media only) - |
| Media+ Organism (Cont.) - |
| 25 % of MBC 63.41 |
| 50% of MBC 71.96 |
| 75% of MBC 79.67 |

Table S1: SeNP Antibiofilm activity against *E. faecalis* and *B. Subtilis* at 25, 50, and 75% MBC

Table S2: SeNP Antibiofilm activity against *S. typhi*, *E.Coli*, and *P. aeruginosa* at 25, 50, and 75% MBC.

| **SeNP - MBC% of** ***S.typhi* Anti-biofilm %** |
| --- |
| Blank (Media only) - |
| Media+Organism (Cont.) - |
| 25% of MBC 82.16 |
| 50% of MBC 88.25 |
| 75% of MBC 92.14 |
| **SeNP - MBC%**  ***E.coli* Anti-biofilm %** |
| Blank (Media only) - |
| Media+Organism (Cont.) - |
| 25 % of MBC 80.43 |
| 50% of MBC 89.44 |
| 75% of MBC 93.47 |
| **SeNP - MBC%**  ***P. aeruginosa* Anti-biofilm %** |
| Blank (Media only) - |
| Media+Organism (Cont.) - |
| 25 % of MBC 54.43 |
| 50% of MBC 74.49 |
| 75% of MBC 90.06 |
